# GABPα Binding to Overlapping ETS and CRE DNA Motifs Is Enhanced by CREB1: Custom DNA Microarrays

**DOI:** 10.1534/g3.115.020248

**Published:** 2015-07-16

**Authors:** Ximiao He, Khund Sayeed Syed, Desiree Tillo, Ishminder Mann, Matthew T. Weirauch, Charles Vinson

**Affiliations:** * Laboratory of Metabolism, National Cancer Institute, National Institutes of Health, Bethesda, Maryland 20892; † Center for Autoimmune Genomics and Etiology, Division of Biomedical Informatics and Division of Developmental Biology, Department of Pediatrics, Cincinnati Children’s Hospital Medical Center, University of Cincinnati College of Medicine, Cincinnati, Ohio 45229

**Keywords:** ETS, CRE, GABPα, CREB1, cooperative DNA binding

## Abstract

To achieve proper spatiotemporal control of gene expression, transcription factors cooperatively assemble onto specific DNA sequences. The ETS domain protein monomer of GABPα and the B-ZIP domain protein dimer of CREB1 cooperatively bind DNA only when the ETS (^C^/_G_CGGAA**GT**) and CRE (**GT**GACGTCAC) motifs overlap precisely, producing the ETS↔CRE motif (^C^/_G_CGGAA**GT**GACGTCAC). We designed a Protein Binding Microarray (PBM) with 60-bp DNAs containing four identical sectors, each with 177,440 features that explore the cooperative interactions between GABPα and CREB1 upon binding the ETS↔CRE motif. The DNA sequences include all 15-mers of the form ^C^/_G_CGGA—–CG—, the ETS↔CRE motif, and all single nucleotide polymorphisms (SNPs), and occurrences in the human and mouse genomes. CREB1 enhanced GABPα binding to the canonical ETS↔CRE motif CCGGAAGT two-fold, and up to 23-fold for several SNPs at the beginning and end of the ETS motif, which is suggestive of two separate and distinct allosteric mechanisms of cooperative binding. We show that the ETS-CRE array data can be used to identify regions likely cooperatively bound by GABPα and CREB1 *in vivo*, and demonstrate their ability to identify human genetic variants that might inhibit cooperative binding.

Cooperative binding of sequence-specific DNA binding proteins is a cornerstone of transcriptional regulation in eukaryotic genomes (Arnosti and Kulkarni 2005; Wunderlich and Mirny 2009; Martinez and Rao 2012; Ptashne 2013, 2014). A classic example is the interferon-beta enhanceosome, in which multiple transcription factors (TFs) bind overlapping and adjacent transcription factor binding sites (TFBS) (Panne *et al.* 2004, 2007). Among these, the ATF-2/c-Jun heterodimer binds the major groove of DNA, and IRF-3 binds in the minor groove of the same DNA base pairs without protein–protein interactions. Instead, it appears that the cooperative binding of these three proteins is via allosteric changes to the DNA (Panne 2008). Another example of overlapping TFBS is the GT dinucleotide within the ETS motif (^C^/_G_CGGAA**GT**) overlapping with the GT dinucleotide in the palindromic CRE motif (**GT**GACGTCAC) to produce the ETS↔CRE motif (^C^/_G_CGGAA**GT**GACGTCAC), which localizes to proximal promoters in mammals (Chatterjee *et al.* 2011; Rozenberg *et al.* 2013). The dimeric B-ZIP protein CREB1 (Vinson *et al.* 2002) enhances binding of the monomeric ETS protein GABPα (Batchelor *et al.* 1998; Garvie *et al.* 2001; Hollenhorst *et al.* 2011a) to the ETS↔CRE motif only when the two motifs are spaced in the configuration shown above (Chatterjee *et al.* 2011). Computational modeling and DNA binding experiments have shown that the GT dinucleotide, which is common to both the ETS and CRE motifs, is bound by the CREB1 dimer in the major groove, and that GABPα binds in the minor groove, without protein–protein interactions between the two proteins (Chatterjee *et al.* 2011). Other B-ZIP proteins, including ATF, Fos, and Jun, are also reported to be interaction partners of ETS proteins (Hai *et al.* 1988; Ooyama *et al.* 1989; Sawada *et al.* 1999).

Protein binding microarrays (PBMs) (Mukherjee *et al.* 2004; Berger *et al.* 2006) are an experimental tool for systematically assaying the DNA binding preferences of TFs. Recently, we and others have used PBMs with custom-designed probe sequences to interrogate the binding specificities of heterodimeric interactions between members of the same TF family (Bolotin *et al.* 2010; Siggers *et al.* 2012; Mann *et al.* 2013; Gordan *et al.* 2013). Here, we investigate the sequence-specific cooperative binding of GABPα and CREB1, two TFs from different structural classes, using custom PBMs containing 177,440 DNA features consisting of the ETS↔CRE motif and variants.

## Materials and Methods

### Cloning and expression of mouse B-ZIP proteins

We obtained a GABPα-GST plasmid from the Tim Hughes laboratory, in which the DNA binding domain of GABPα is fused to Glutathione S-transferase (GST) at the C-terminal end to produce the chimeric protein GABPα-GST (Badis *et al.* 2009). The CREB1 B-ZIP domain without GST was expressed from a pT5 plasmid (Ahn *et al.* 1998). The proteins were expressed by *in vitro* translation system (IVT) reactions using PURExpress In Vitro Protein Synthesis Kit (NEB) as described in Badis *et al.* (2009). For the GABPα-GST IVT reaction, 2.5 ng, 8 ng, or 30 ng of plasmid was added to 25 ul of IVT solution. For analysis of cooperativity between GABPα-GST and CREB1, equal amounts of plasmids (2.5:2.5, 8:8, or 30:30 ng) were used in IVT reactions in a final volume of 25 ul. IVT reactions were performed at 37° for 2 hr, and then 23 ul of the IVT solution was added to the arrays.

### PBM experiments

The single-stranded DNA 60-mer ETS↔CRE microarrays were made double-stranded by primer extension as described previously (Badis *et al.* 2009). The protein binding reactions were also performed as previously described (Badis *et al.* 2009). Protein-bound microarrays were scanned to detect Alexa Fluor 647-conjugated anti-GST using at least two different laser power settings to capture a broad range of feature intensities and to ensure signal intensities were below saturation for all spots. Microarray images were analyzed using ImaGene (BioDiscovery Inc.), bad spots were manually flagged, and the extracted data were used for further analysis. All proteins in this study were assayed twice, with high agreement between replicates (R = 0.92–0.98; Supporting Information, Figure S1). The data are available at the NIH public ftp site (ftp://helix.nih.gov/pcf/chuck/Array/).

### Examination of cooperative binding of GABPα and CREB1 *in vivo*

We identified five cell lines from the ENCODE consortium (https://www.encodeproject.org/) with ChIP-seq data available for both GABPα and CREB1 (A549, GM12878, H1hESC, HepG2, and K562). For each cell line, we divided the GABPα ChIP-seq peaks into two groups based on the presence of CREB1 binding: “GABPα +CREB1” (regions bound by both GABPα and CREB1) and “GABPα −CREB1” (GABPα peaks that do not overlap CREB1 binding peaks).

For each of these two groups, we calculated an enrichment score for the ETS consensus motif and each of its 1-bp variations, relative to occurrences in the human genome [build hg19, downloaded from the UCSC genome browser (http://genome.ucsc.edu/)]. For each motif *M* with length *L* [for the consensus ETS motif (CCGGAAGT), *L* = 8], we denote *M* (*x_start_:x_end_*) to record the genomic positions where the motif starts and ends: *x_1_:x_1_+L−1*, *x_2_: x_2_+L*-*1...x_N_: x_N_+L*-*1*, with *N* being the total number of motifs in the human genome. For each position *x_i_: x_i_+L-1*, if it overlapped with the given group, then *x_i_* = 1; otherwise, *x_i_* = 0. For each group, the observed (*OCC_obs_*) and expected (*OCC_exp_*) occurrences of the motif were calculated as: $OCC_{obs}=\overset{N}{\underset{i=1}{\sum }}x_{i}$ and $OCC_{exp}=N\times \frac{L_{r}}{L_{g}}$, where *N* is the total number of motifs in the whole genome, *L_r_* is the total length of base pairs in the group, and *L_g_* is the total length (in base pairs) of the human genome. The enrichment score (*E*) for motif *M* was calculated as $E=\frac{OCC_{obs}}{OCC_{exp}}$, where *OCC_obs_* is the observed occurrences and *OCC_exp_* is the expected occurrence of motif *M* in the group. We calculated the motif enrichment score in both groups, denoted as *E_GABPα + CREB1_* and *E_GABPα - CREB1_*.

We identified regions of the genome likely to be cooperatively bound by GABPα and CREB1 by scanning for ETS/CRE variants in the “GABPα + CREB1” ChIP peak groups using models derived from our arrays. We created position frequency matrix (PFM) DNA binding models from the ETS-CRE array probe intensities using the SNP portion of the arrays. Briefly, we first identified the sequence with the highest median signal intensity across its 40 probes, *S_max_*. All other possible SNPs were then assigned a relative score to *S_max_* by dividing their median signal intensity by *S_max_*. A PWM was then constructed from these values. For a given position in the PWM, the nucleotide with the highest median intensity was assigned a value of 1. Each of the other nucleotides were then assigned values corresponding to their relative score. This PWM was then converted into a PFM by summing the values at each position, and dividing each value by this sum, such that the values at each position sum to 1.

We created two sets of PFMs: those derived from experiments where both GABPα and CREB1 were present (using signals from the array experiments with relative concentrations of 2.5:2.5, 8:2.5, 8:8, and 30:30) and experiments where only GABPα was present (concentrations 2.5:0, 8:0, 30:0, and 100:0), supplemented with other *in vitro*–derived PFMs for GABPα and CREB1 obtained from the CisBP database (Weirauch *et al.* 2014). These PFMs were used to scan all sequences in each ChIP peak group using the energy scoring system used by BEEML (Zhao *et al.* 2009). The relative ranking of each sequence was then compared between PFMs derived from arrays with and without CREB1. Sequences assigned a top rank by models derived from the “GABPα and CREB1” arrays, but middle-to-low ranks by models from the “single protein” arrays are likely to be bound cooperatively by both proteins *in vivo*, according to our *in vitro* models. We identified such sequences using a false discovery rate (FDR)-based approach. For the peak sequences of each ChIP-seq dataset, we created a matched scrambled sequence set by permuting each sequence while maintaining dinucleotide frequencies. We then scored these scrambled sequences with a given PFM (as described above) and combined these sequences and scores with the "real" ChIP-seq sequences and scores. We sorted the list by PFM score and then calculated the FDR as a function of the PFM score as the fraction of sequences exceeding the given cutoff that are members of the scrambled set.

### Data availability

GABPa and CREB1 plasmids are available upon request. The data for protein binding microarrays (PBM) are available at the NIH public ftp site (ftp://helix.nih.gov/pcf/chuck/Array).

## Results

### Design of 177,440 feature ETS-CRE Agilent custom DNA microarray

We designed an Agilent DNA microarray, named the ETS-CRE array, containing four identical sectors, with each sector having 177,440 single-stranded DNA 60-mers containing the ETS↔CRE motif and variants (Table 1). The 60-mer containing the ETS↔CRE motif on the ETS-CRE array has the ETS motif toward the solution (solution-GTCCTCAAGA **^C^/_G_CGGAAGTGACGTCAC**GACTCAGGTG|GGACACACTTTAACACATGGAGAG-slide). The variants are of three basic categories: variants of the core motif; single nucleotide polymorphisms (SNPs); and genomic occurrences (Table 1). The core variants include all 15-mers of the form ^C^/CGGA—–CG—(features 1–65,536) and /_G_CGGA—–CG—(features 65,537–131,072), with the eight "-"s indicating variable positions. The SNP category contains 40 probes each for both 16-mer ETS↔CRE motifs (^C^/_G_CGGAAGTGACGTCAC) (Wei *et al.* 2010) and 40 probes for each SNP (features 131,073–134,992). The “genomic” probes contain 36 bps covering all occurrences of———–^C^/_G_CGGA—–CG—— —– from the mouse genome (features 134,993–163,391) and a subset from human (features 163,392–177,440). The 24-bp (GGACACACTTTAACACATGGAGAG) near the slide is constant and complementary to the DNA primer used for double-stranding the DNA prior to the binding experiment (see *Materials and Methods*).

**Table 1:** Design of Agilent microarray containing 177,440 features

Category	Description	Probe IDs	Count
**ETS Core Variants**	CCGGA—–CG—(65,536)	1–65,536	65,536
	GCGGA—–CG—(65,536)	65,537–131,072	65,536
**ETS↔CRE SNPs**	ETS↔CRE CC motif:CCGGAAGTGACGTCAC × 40	131,073–131,112	40
	ETS↔CRE CC motif SNPs: 1-bp variants (SNPs) of CCGGAAGTGACGTCAC (3*16) × 40	131,113–133,032	1920
	ETS↔CRE GC motif: ETS-CRE motif (GCGGAAGTGACGTCAC × 40)	133,033–133,072	40
	ETS↔CRE GC motif SNPs: 1-bp variants (SNPs) of GCGGAAGTGACGTCAC (3*16) × 40	133,073–134,992	1920
**Genomic Occurrences**	All unique 36-mer with CCGGA—–CG extracted from mouse (UCSC mm9)	134,993–150,152	15,160
	All unique 36-mer with GCGGA—–CG extracted from mouse (UCSC mm9)	150,153–163,391	13,239
	Randomly selected unique 36-mer with CCGGA—–CG extracted from human (UCSC hg19)	163,392–170,391	7000
	Randomly selected unique 36-mer with GCGGA—–CG extracted from human (UCSC hg19)	170,392–177,440	7049

### GABPα-GST binding to ETS-CRE array

**Figure 1:**
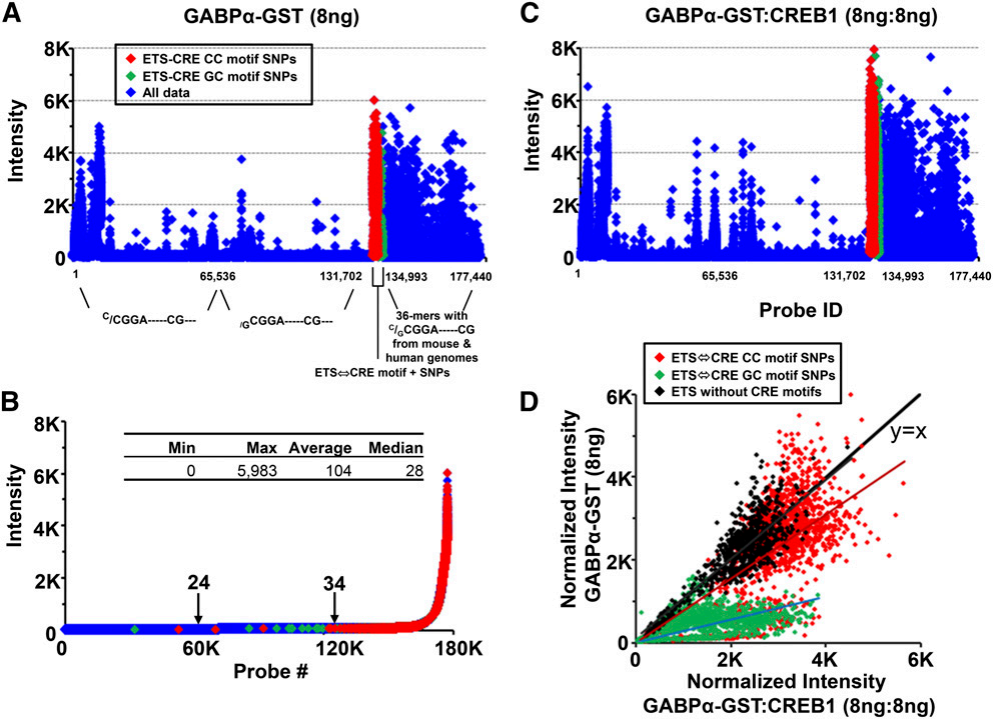
Binding activity of GABPα to the ETS-CRE array. (A) GABPα-GST binding (8 ng) to 177,440 features on the ETS-CRE array presented sequentially as outlined in Table 1. (B) GABPα-GST binding to 177,440 features arranged by fluorescence intensity with the ETS↔CRE motifs and SNPs colored as in (A). Arrows on the plot indicate the fluorescence intensities observed at the 60Kth and 120Kth ranked probes on the array. (C) GABPα-GST binding to 177,440 features on the ETS-CRE array in the presence of CREB1 (8 ng:8 ng) presented sequentially as outlined in Table 1. Data points are colored as in (A). (D) Scatterplot comparing the fluorescence intensities from ETS-CRE array experiments obtained from GABPα-GST alone (y-axis) and GABPα-GST + CREB1 (x-axis).

We monitored a chimeric protein containing the ETS domain of GABPα fused to GST (GABPα-GST) binding to the ETS-CRE array using a fluorescent antibody against GST (Figure 1) (Badis *et al.* 2009). GABPα-GST binding to the 177,440 features on the ETS-CRE array is presented sequentially as outlined in Table 1, with robust binding near feature 131,000, which contains the canonical ETS↔CRE motif CCGGAAGTGACGTCAC 16-mer and SNPs (Figure 1A). Figure 1B presents GABPα-GST binding to all 177,440 features ordered by fluorescence intensities with features containing the canonical ETS↔CRE motifs and SNPs highlighted. Half of the probes have fluorescence intensity values less than the SNPs, even though all the features contain the core ETS motif, indicating that most of the SNPs retain some degree of binding.

### CREB1 enhances GABPα-GST binding to the ETS-CRE array

We next monitored GABPα-GST binding at three concentrations (2.5 ng, 8 ng, and 30 ng) to the ETS-CRE array in the absence and presence of the CREB1 B-ZIP domain without GST, allowing us to examine GABPα-GST binding without the complication of also monitoring CREB1 binding (as would occur if CREB1 was also GST-tagged). In the presence of CREB1, we observed increased GABPα-GST binding, with a qualitatively similar overall pattern to the binding of GABPα-GST alone (Figure 1C). To exclude potential biases between the sectors, we also normalized the fluorescence intensities based on the probes that contain only GABPα sites, not CREB1 sites. Figure 1D shows the normalized fluorescence intensities of GABPα-GST for all probes with or without CREB1. There is an increase in fluorescence intensity for many probes when CREB1 is added, with no dramatic decrease in binding to any probes (*i.e.*, there are very few points in the upper left quadrant), suggesting that CREB1 only enhances the binding of GABPα-GST.

It is important to evaluate whether the binding of GABPα-GST is saturating to allow for an accurate evaluation of the effect of CREB1 on GABPα-GST binding. Western blots of the IVT reaction indicate a correspondence between the concentration of input plasmid to GABPα protein concentration, which is saturated by 100 ng (Figure S2). Moreover, the concentration of GABPα in GABPα+CREB1 mixtures is lower than the observed concentration of GABPα only, indicating that the increase of GABPα binding in GABPα+CREB1 mixtures in the array is not due to the increased GABPα concentration (Figure S2). On the same slide, we added an IVT primed with a low concentration of GABPα-GST plasmid (2.5 ng) with or without CREB1 (2.5 ng and 8 ng) and a sector with an IVT primed with 12-times more GABPα-GST plasmid (30 ng). Figure S3 shows the fluorescence intensities for the 177,440 features in four sectors. At both concentrations, CREB1 increased GABPα-GST binding, but this enhancement is less than that observed for GABPα-GST binding at high concentrations (30 ng), indicating that GABPα-GST binding at 2.5 ng to the canonical motif is not saturating.

### GABPα-GST binding to ETS↔CRE motif and SNPs

**Figure 2:**
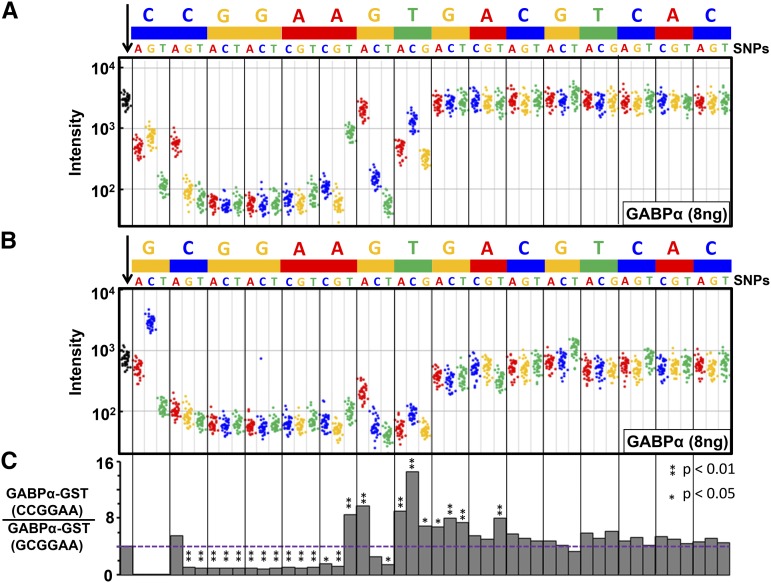
GABPα binding to SNPs in two ETS↔CRE motifs. (A) GABPα-GST binding to 1960 features containing the ETS↔CRE 16-mer CCGGAAGTGACGTCAC and 48 SNPs on the ETS-CRE array. The first column of the figure contains 40 black spots representing GABPα-GST binding to the 40 features containing the consensus ETS↔CRE motif CCGGAAGTGACGTCAC. The rest of the columns represent 40 features for each of the 48 SNPs, as indicated. (B) GABPα-GST binding to 1960 features containing the weaker ETS↔CRE 16-mer GCGGAAGTGACGTCAC and 48 SNPs on the ETS-CRE array. (C) Histogram of the ratio of binding to the strong (**C**CGGAA) and weak (**G**CGGAA) ETS motif and SNPs. Horizontal dashed line indicates the ratio of binding to the strong (**C**CGGAA) and weak (**G**CGGAA) consensus ETS motif. Asterisk (*) indicates if the fold change is statistically significant when compared to the fold change of the strong motif (CCGGAA) (*P < 0.05; **P < 0.01).

We next examined GABPα-GST binding to the 40 replicate features (replicates) for the canonical ETS↔CRE motif (^C^/CGGAAGTGACGTCAC) and the 40 features (replicates) for each of the 48 SNPs (Figure 2). The variation in binding within the 40 features containing an identical DNA sequence is approximately two-fold (Figure 2A). GABPα-GST binds more strongly to the canonical ETS↔CRE motif (CCGGAAGT) than to any SNP. SNPs in the ETS core GGA trinucleotide (CC**GGA**AGT) uniformly decreased binding up to 27-fold. GABPα-GST binding to the SNPs for the five bases in bold (**CC**GGA**AGT**) at the beginning and end of the ETS motif is variable, consistent with known degeneracy in the GABPα recognition motif at these positions in motifs derived from *in vitro* (Badis *et al.* 2009) and *in vivo* (Valouev *et al.* 2008) data. There is no simple relationship between binding to SNPs based on their nucleotide type (*i.e.*, whether they are purines or pyrimidines).

GABPα-GST binds four-fold weaker to the ETS↔CRE motif starting with guanine (/_G_CGGAAGTGACGTCAC) (Figure 2B) (Wei *et al.* 2010). To evaluate whether SNPs have different properties in the two ETS motifs, we examined the ratio of binding to the SNPs in the two versions of the ETS motif (Figure 2C). SNPs with a ratio significantly greater (*P* < 0.05, Fisher’s exact test) than four (the value of four being the ratio of binding to the strong ^C^/ motif *vs.* the weak /_G_ motif) are more deleterious to binding in the context of the weaker (/_G_) motif. All such SNPs are located close to or within the CRE motif. For example, the 3′ T→C SNP (CCGGAAG**T** → CCGGAAG**C**), marked with an asterisk (*) in Figure 2C, is better bound in the context of the ^C^/ motif than in the context of the /_G_ motif (GCGGAAG**T** → GCGGAAG**C**). For ratios significantly less than four (*P* < 0.05), the SNP is more deleterious in the context of the stronger ^C^/ motif. However, all SNPs with ratios less than four have intensities close to the background for both versions of the motif, making it difficult to evaluate if differences in binding are occurring.

### CREB1 preferentially enhances GABPα-GST binding to two groups of SNPs in the ETS↔CRE motif

**Figure 3:**
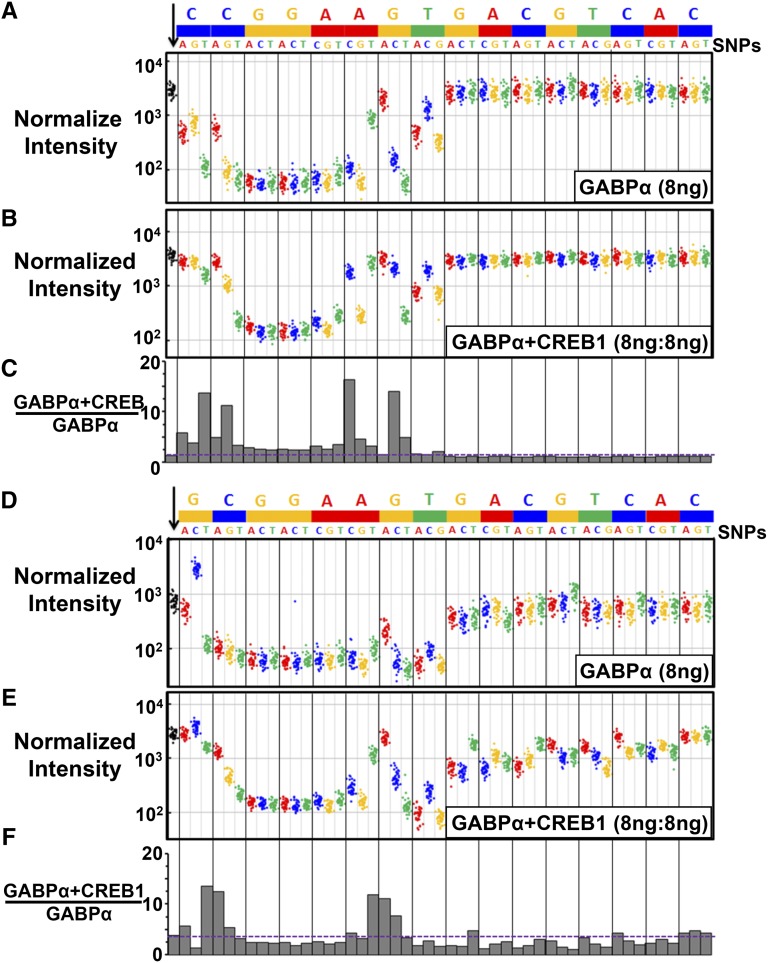
CREB1 enhances GABPα binding to several SNPs in the ETS↔CRE motif. (A) GABPα-GST (8 ng) binding to 1960 features containing the ETS↔CRE 16-mer CCGGAAGTGACGTCAC and 48 SNPs on the ETS-CRE array. The first column of the figure contains 40 black spots representing GABPα-GST binding to the 40 features containing the consensus ETS↔CRE motif CCGGAAGTGACGTCAC. The rest of the columns represent 40 features for each of the 48 SNPs, as indicated. (B) The normalized GABPα-GST binding in the presence of equal concentration (8 ng:8 ng) of the CREB1 plasmid on the ETS-CRE array. (C) Histogram of the ratio of GABPα-GST array intensities ± CREB1 to the consensus and SNP probes. Horizontal dashed line indicates the ratio of GABPα+CREB1/GABPα binding to the consensus motif. (D–F) Same as in (A–C), but for GABPα-GST (± CREB1) binding to 1960 features containing the weaker ETS↔CRE 16-mer GCGGAAGTGACGTCAC motif and 48 SNPs on the ETS-CRE array.

CREB1 increased binding of GABPα-GST to the canonical motif (^C^/) approximately two-fold (*P* < 0.0001, *t*-test). CREB1 also increases binding to all SNPs, although none is more strongly bound than the canonical motif. In particular, CREB1 increased GABPα-GST binding to the weakly bound SNPs in the core GGA trinucleotide three-fold (Figure 3, A–C). CREB1 has variable effects on increased GABPα-GST binding for two groups of SNPs in bold (**CC**GGA**AGT**) at the beginning and end of the ETS motif, with increases of up to 20-fold for four SNPs, **T**CGGAAGT, C**G**GGAAGT, CCGGA**C**GT, and CCGGAA**C**T. Importantly, these are the same SNPs that strongly reduced GABPα-GST binding in the absence of CREB1. There is a nonlinear relationship between GABPα array intensities and cooperativity (ratio of GABPα+CREB/GABPα; Figure S4), suggesting that the increased cooperativity by CREB1 is not simply due to a decrease in affinity of the GABPα monomer site. SNPs localized within the CREB1 motif caused only a slight decrease in CREB1-dependent GABPα-GST binding, suggesting that these SNPs instead act by decreasing CREB1 binding, as expected. Similar results were obtained at two other GABPα-GST and CREB1 concentrations (Figure S5 and Figure S6), but in the concentration of 2.5 ng of GABPα-GST and GABPα-GST+CREB1 there is a clear decrease in binding to SNPs localized within the CREB1 motif (Figure S5, C and F), which indicate the binding ability of GABPα depends less on the changes of CREB1 binding when GABPα concentrations are high.

CREB1 preferentially enhances GABPα-GST binding to SNPs in the weaker (/_G_) ETS motif in the same two regions of the ETS motif seen for the stronger motif. However, the particular SNPs bound better by GABPα-GST in the presence of CREB1 are different for the two ETS↔CRE motifs (Figure 3, D–F). At the beginning of the ETS motif, CREB1 increases GABPα-GST binding to C**G**GGAAGT approximately 16-fold, but to G**G**GGAAGT only approximately eight-fold (Figure 3, C and F). Preferential binding to SNPs in the G version of the motif are also observed, *e.g.*, binding to G**A**GGTTGT is enhanced approximately 17-fold but C**A**GGTTGT is enhanced only approximately seven-fold. SNPs at the end of the ETS motif also show variable effects. For example, CREB1 increased GABPα-GST binding to the motif CCGGAA**C**T 20-fold, but for GCGGAA**C**T the increase is only eight-fold. Conversely, binding to the motif GCGGAA**A** was increased 16-fold, whereas binding to CCGGAA**A** was only increased two-fold. SNPs to the CRE portion of the ETS-CRE motif in **bold** (/_G_CGGAAG**TGACGTCAC**) have more specific effects on CREB1-dependent GABPα-GST binding than SNPs in ^C^/CGGAAG**TGACGTCAC**. In summary, we observe two distinct regions of the ETS motif that are differentially bound in the presence of CREB1, and the specific SNPs affecting the binding are related but distinct, depending on the presence of a C or G at the 5′ end of the ETS motif.

### Comparison of the ETS-CRE custom array to universal PBMs

**Figure 4:**
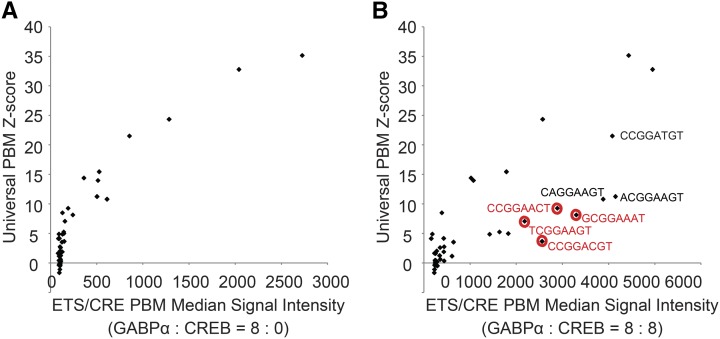
Comparison between universal and custom PBMs. Comparison of custom ETS-CRE array median signal intensity (x-axis) and universal PBM Z-scores (y-axis) for each of 46 possible 8 base variants of the ETS motif. (A) Results using ETS-CRE arrays in the absence of CREB1 (8 ng GABPα, 0 ng CREB1) *vs.* Z-scores obtained from GABPα binding to universal PBMs. (B) Results using ETS-CRE arrays in the presence of CREB1 (8 ng GABPα, 8 ng CREB1). Consensus GABPα sequence is indicated in black. 8-mers highlighted in red demonstrate high differential cooperativity ± CREB1 (*i.e.*, they had a >10-fold increase in binding in Figure 3C).

We next compared the results of our custom PBM arrays to those obtained using universal PBM (uPBM) array designs, which contain 32 occurrences of all nonpalindromic 8-mers on arrays containing ∼40,000 probes (Wei *et al.* 2010). Binding affinities for a given TF on the universal PBM arrays are represented as Z-scores, which are an aggregate value for binding to all 32 occurrences of each 8-mer on the PBM. In the absence of CREB1, GABPα-GST binding to the 48 SNPs shows highly similar results on the two platforms (Figure 4A). This result highlights that the uPBM design accurately measures changes in binding for SNPs for motifs that are 8-mers. However, when CREB1 is added to the ETS-CRE arrays, the binding of GABPα-GST to several SNPs is greatly altered (Figure 4B), and these results are similar at the other protein concentrations examined (Figure S7). Notably, the 8-mer sequences that show the largest increase in binding in the presence of CREB1 (*i.e.*, the points that shift the farthest to the right in Figure 4B) are generally the same SNPs we observed in Figure 3.

### ETS-CRE arrays identify sites bound cooperatively by GABPα and CREB1 *in vivo*

**Figure 5:**
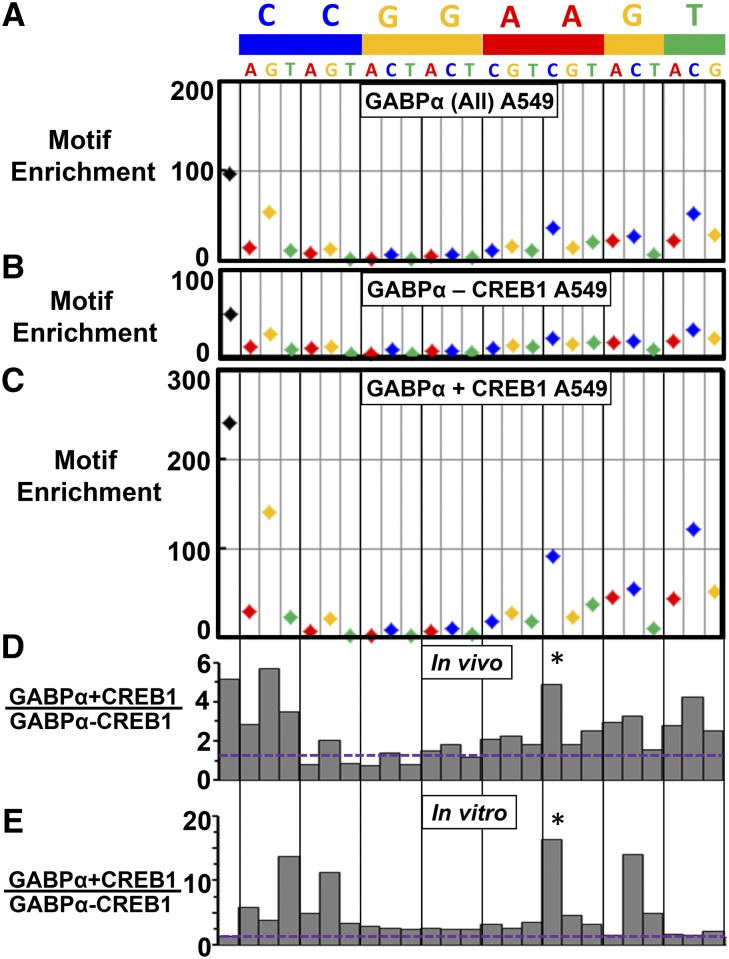
CREB1 enhances GABPα binding to SNPs identified by the ETS-CRE array and in genomic regions cobound by CREB1 and GABPα. (A) Enrichment of ETS 8-mer consensus and each 1-bp variation in GABPα ChIP-seq peaks in A549 cells. (B and C) Enrichment of ETS 8-mer consensus and each 1-bp variation in GABPα ChIP-seq peaks in A549 cells that (B) do not overlap CREB1 ChIP-seq peaks (GABPα - CREB1) and (C) do overlap CREB1 ChIP-seq peaks (GABPα + CREB1). (D) Histogram of the ratio of motif enrichment ± CREB1 to the consensus and 1-bp variations of the ETS motif (CCGGAAGT) in A549 cell lines. (E) The *in vitro* data shown here are identical to that in Figure 3C (the first 8 bases) and are included here for ease of comparison. Asterisk (*) indicates SNP highlighted in the text.

To examine if the differences in GABPα-GST binding to the ETS motif and SNPs observed *in vitro* are relevant biologically, we next asked if the sequences cooperatively bound by GABPα and CREB1 on the ETS-CRE arrays are also colocalized *in vivo*. To this end, we obtained ENCODE ChIP-seq data from five cell lines where the genomic localization of both GABPα and CREB1 were assayed and computed the occurrence and enrichment of the ETS 8-mer motif (CCGGAAGT, see *Materials and Methods*). For example A549 cells contain 10,940 GABPα ChIP-seq peaks representing 0.2% of the human genome. The genome has 8608 occurrences of the canonical ETS 8-mer, and 1645 (19.1%) of motif occurrences are present in GABPα binding peaks, resulting in a 96-fold enrichment (Figure 5A, Table S1). The canonical ETS motif is more enriched than any of its SNP variants. SNPs that alter the CG dinucleotide are nearly 10-fold more abundant in the genome and often occur in GABPα peaks. For example, C**A**GGAAGT has 88,465 occurrences in the genome and 1406 are bound, even though enrichment is relatively lower at 8.8-fold. Several additional SNPs that are well bound *in vitro* are also bound *in vivo*, highlighting that DNA binding specificity is important for GABPα peak localization, with similar trends observed in the remaining four cell types (Table S1 and Table S2).

We next examined if GABPα and CREB1 colocalization preferentially occurs at the same SNPS where CREB1 facilitated GABPα binding *in vitro*. In regions where GABPα and CREB1 ChIP-seq peaks overlap (which we denote as GABPα+CREB1) in A549 cells, the consensus ETS motif is five-fold more enriched (*P* < 0.0001, Fisher’s exact test) relative to GABPα peaks where CREB1 is absent (GABPα-CREB1; Figure 5, B–D). We also observe enrichment of specific SNPs in GABPα+CREB1 peaks, a pattern similar to what is observed on the ETS-CRE arrays, providing evidence that preferential GABPα binding to these sequences occurs only when CREB1 is colocalized *in vivo*. For example, CCGGA**C**GT is the second most enriched SNP sequence in GABPα+CREB1 peaks in A549 cells (Figure 5D, marked with an asterisk), and it is also the most enhanced SNP observed in our *in vitro* PBM experiments (Figure 5E). In h1ESC cells, we observe intermediate levels of enrichment of the ETS motif and SNPs within overlapping GABPα+CREB1 peaks (Figure S8, A–C). In other cell types (GM12878, HepG2, and K562 cells), CREB1 contributes relatively little to GABPα binding (∼1.5-fold increase to the consensus motif and SNPs in GABPα+CREB1 peaks relative to GABPα-CREB1 peaks; Figure S8, D–L), suggesting that CREB1 is not as active in these cell types even though it is bound to DNA. It is likely that additional molecular events need to occur to facilitate cooperative binding between GABPα and CREB1, such as post-translational modifications that are known to be needed for CREB1 to recruit coactivators (Chrivia *et al.* 1993).

### The ETS-CRE array identifies genomic loci that are cooperatively bound by GABPα and CREB1

**Figure 6:**
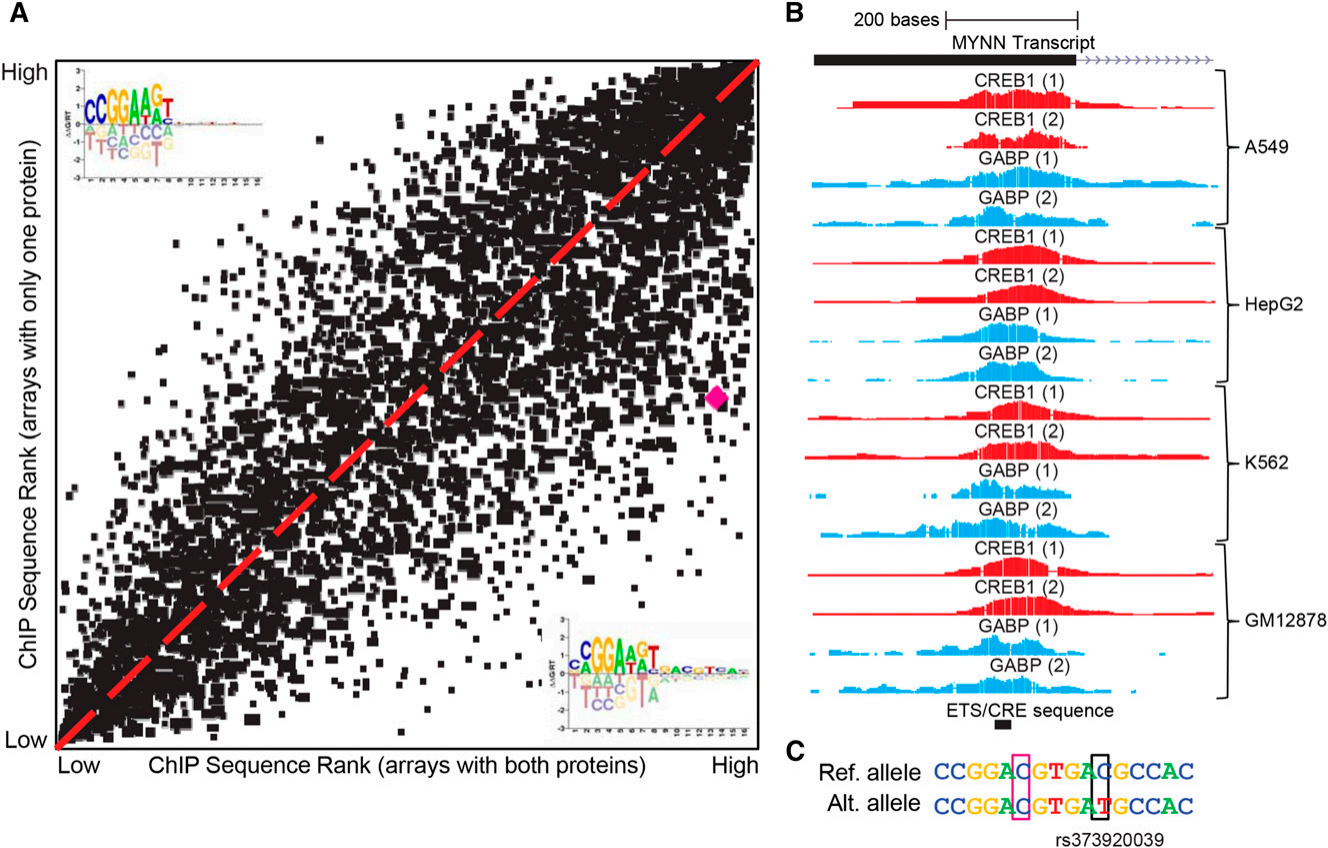
The ETS-CRE array identifies genomic sequences specifically cobound by CREB1 and GABPα. (A) Cooperative binding to cobound genomic sequences. Position weight matrix (PWM) DNA binding models were created from PBM probe intensities based on (1) experiments performed with both GABPα and CREB1 and (2) experiments performed with only one of these proteins (see *Materials and Methods*). These PWMs were used to score sequences bound by both CREB1 and GABPα, based on ChIP-seq experiments. The scatterplot depicts, for each sequence, the highest score obtained by any PWM trained on PBM experiments performed in the presence of both GABPα and CREB1 (x-axis) or using only a single protein (y-axis). Results are shown for A549 cells and are representative of the other four cell types with ChIP-seq data available for both TFs (data not shown). Magenta data point indicates an example (further highlighted in B) of a sequence that scores well on arrays in the presence of both proteins, but scores poorly using models derived from only one of the proteins (and, hence, likely contains a cooperative sequence). Sequence logos depicting representative PWMs are shown for GABPα+CREB1 (lower right) and GABPα only (upper left). (B) Genomic context of cooperative binding example highlighted in (A). UCSC Genome Browser snapshot depicts (top to bottom): 5′ UTR and first intron of *MYNN* gene, signal of ChIP-seq replicates for CREB1 (red) and GABPα (blue) in four cell lines (with replicates), and the location of the putative cooperative ETS↔CRE element (black box). (C) Zoom-in view of putative ETS↔CRE from (B). Pink box indicates the “C” sequence detected in our arrays that is only tolerated in the presence of CREB1. Black box indicates a human genomic SNP with an alternative allele that would likely disrupt cooperative binding of GABPα and CREB1.

We next asked if we could use data from the ETS-CRE arrays to identify specific genomic loci where GABPα and CREB1 are cooperatively binding. To this end, we created position weight matrix (PWM) DNA binding models from each of our custom PBM experiments and used them to score all genomic sequences bound by both GABPα and CREB1 in the same cell type (*i.e.*, GABPα+CREB1 peaks; see *Materials and Methods*). The scatterplot in Figure 6A depicts, for each of the 5776 genomic regions co-bound by both GABPα and CREB1 in A549 cells, the best binding score predicted by any PWM derived from our custom PBM experiments that included CREB1 (x-axis), and the best score predicted by any PWM derived from experiments using only one of the two proteins (y-axis). Representative PWMs are depicted in the corners of the plot and closely reflect the known GABPα motif (upper left) and the new, cooperative GABPα+CREB1 binding motif depicted in Figure 2. As expected, many sequences fall along the Y = X diagonal. Such sequences likely represent cases in which GABPα is binding independently of CREB1 (*i.e.*, they could be predicted equally well with cooperative and noncooperative models). Notably, the majority of points fall in the lower right quadrant of the plot. These points represent sequences that could only have been predicted using data from experiments performed in the presence of both GABPα and CREB1, and hence might represent examples of *in vivo* cooperative binding. We created high-confidence sets of likely cooperative sequences by restricting to those with false discovery rates (FDRs) less than 5% in at least one GABPα+CREB1 dataset and an FDR >15% in every GABPα and CREB1 monomeric PWM dataset (see *Materials and Methods*). Seventy-eight such sequences were identified in the A549 ChIP-seq dataset, and an example of one is illustrated as a magenta point in Figure 6A. This sequence has a best rank of #291 based on PWMs derived from arrays with GABPα+CREB1 among all 5776 ChIP-seq co-bound sequences but only has a best ranking of #2841 based on PWMs derived from arrays assaying GABPα or CREB1 in isolation. Figure 6B depicts the genomic context of this sequence, which is located within the 5′ UTR of the *MYNN* gene, and is bound by both GABPα and CREB1 in four cell lines. The ETS↔CRE sequence is located in the middle of the ChIP-seq peaks (bottom of Figure 6B), concordant with the idea that it is likely bound by both proteins. Figure 6C shows the ETS/CRE sequence in detail. The purple box indicates the cooperative “C” nucleotide that is bound *in vitro* only in the presence of CREB1, as highlighted in Figure 5, D and E. Interestingly, this sequence also contains a genomic SNP (rs373920039) that would likely disrupt the binding of GABPα and CREB1 by altering the CRE sequence (black box, Figure 6C), highlighting the potential importance of cooperative binding from a population and disease genetics perspective. Collectively, these results demonstrate that the cooperativity we observe *in vitro* is also likely important *in vivo*, and illustrate the utility of our custom arrays to identify genomic regions containing cooperative binding events that might be disrupted by specific genetic variants.

## Discussion

We examined DNA binding of the ETS domain of GABPα (GABPα-GST) in the absence and presence of the B-ZIP domain dimer of CREB1 to the ETS-CRE array, a custom Agilent microarray, to explore cooperative binding to the ETS↔CRE motif (^C^/_G_CGGAAGTGACGTCAC) and variants. As expected, SNPs to the 8-bp ETS motif decreased GABPα-GST binding. All SNPs to the ETS motif core GGA trinucleotide are poorly bound and all form hydrogen bonds with the ETS protein domain (Hollenhorst *et al.* 2009). SNPs to the 5 bp in bold (**CC**GGA**AGT)** had variable effects on GABPα-GST binding. The SNPs have different contributions to binding in the strong (^C^/) or weak (/_G_) ETS motifs, emphasizing the complexity of these interactions. GABPα-GST in the presence of CREB1 binds the consensus motif CCGGAAGT two-fold better, which is stronger than binding to any of the SNPs. Importantly, CREB1 increases GABPα-GST binding to some SNPs 20-fold, effectively ameliorating the poor binding of GABPα-GST to these SNPs.

Pairs of transcription factors (TFs) can cooperatively bind to DNA directly (Martinez and Rao 2012) or indirectly by competition with nucleosomes (Polach and Widom 1996; He *et al.* 2013). Classical models of cooperative TF binding involve direct protein–protein interactions between TFs to achieve binding specificity, as exhibited by the λ phage repressor (Johnson *et al.* 1979). The full-length λ repressor is composed of two domains, one required for DNA binding and a second that mediates protein–protein interactions between repressor dimer pairs. This dimer interaction is required for the repressor to cooperatively bind adjacent sites on the same face of DNA, which can be separated by distances up to six turns of the DNA helix (Hochschild and Ptashne 1986; Bell *et al.* 2000). In the case of overlapping TFBS, two mechanisms of cooperative binding have been observed. In one case, the DNA binding domains form protein–protein interactions that facilitate cooperative DNA binding, as is vividly illustrated in the cocrystal structure of PU.1 and IRF-4 (Escalante *et al.* 2002). In the second case, TFs cooperatively bind overlapping TFBS and do not form protein–protein interactions, as observed in the beta enhanceosome. Here, cooperative binding is hypothesized to occur through complementarity of DNA conformation at overlapping sites (Panne *et al.* 2007). In other words, complementary conformations promoting cooperative binding between TF pairs are brought about due to sequence-dependent structural changes in DNA at composite sites.

The cooperative binding of GABPα-GST and CREB1 to the ETS↔CRE motif is modeled to be analogous to the enhanceosome. In both cases B-ZIP dimers are involved and there are no direct interactions between the two domains (Chatterjee *et al.* 2011). Analysis of the SNP probes of our ETS-CRE array identified two distinct groups that exhibit more cooperative binding than the canonical motif. One group of SNPs overlaps the ETS and CRE motifs, suggesting that this mechanism of cooperative binding works through sequence-dependent DNA structural changes that allow formation of complementary DNA conformations at overlapping sites (Panne *et al.* 2007). Crystal structures of GABPα and CREB1 binding independently and together to the SNPs with the greatest cooperative binding may provide an experimental opportunity to directly evaluate if complementarity of DNA conformation at overlapping sites can be observed. The second group of SNPs is at the beginning of the ETS motif, 5 bp away from the overlap between the ETS and CRE motif. The mechanism for this type of cooperatively is potentially different and may be better described through molecular dynamics. Here, the binding of CREB1 might stabilize the DNA, which facilitates GABPα-GST binding. The SNP dependence of this potential mechanism highlights the subtlety of cooperative binding (Cooper and Dryden 1984; Harris *et al.* 2001; Kim *et al.* 2013).

ETS TFs interact with TFs from several families to bind sequences containing chimeric aspects of each binding site (Wunderlich and Mirny 2009; Hollenhorst *et al.* 2011b). GABPα initially was observed interacting with GABPβ (another ETS protein) to bind a composite element (Batchelor *et al.* 1998). Forkhead proteins interact at the 5′ end of the ETS motif (De Val *et al.* 2008) while SRF, PAX, and CREB1 interact at the 3′ end of the ETS motif (Hollenhorst *et al.* 2011b). Likewise, many B-ZIP TFs bind to composite elements containing multiple TF families. For example, the cytokine RANTES (regulated upon activation, normal T cell expressed) is induced by LPS through promoter binding by ATF and Jun proteins to a composite site containing nonoverlapping ETS and CRE motifs (Boehlk *et al.* 2000), and BATF and IRF4 bind cooperatively to promote gene activation and T-helper 17 cell differentiation (Glasmacher *et al.* 2012). Here, we show how the complex interactions between two TFs from different families can be interrogated using custom DNA binding microarrays. We note that these interactions cannot be examined using standard universal PBM array designs, because they usually involve composite motifs that are much longer than the motifs that can be assayed using standard arrays. The ETS-CRE custom array design can be used to examine cooperative binding of pairs of additional ETS and B-ZIP family members. Further, additional custom arrays can be designed containing any combination of TF binding elements.

Genome-wide association studies (GWAS) have implicated SNPs in many pathologies. A substantial proportion of these SNPs (85–93%) are noncoding and are thought to act by affecting TF binding sites (Hindorff *et al.* 2009; Maurano *et al.* 2012). The SNP probes of our arrays will be particularly valuable in evaluating whether a disease-associated or trait-associated SNP alters the binding of specific TFs. For example, our data indicate that CREB1 preferentially facilitates GABPα-GST binding to different SNPs within the two ETS motif variants (^C^/CGGAAGT and /_G_CGGAAGT), highlighting the detail in these data. A more elaborate exploration including two and three base pair variants of a canonical motif may yield even more information on evaluating GWAS-identified SNPs. We anticipate that custom-designed PBMs such as the ETS-CRE array will therefore aid not only in understanding the mechanisms underlying TF interactions but also in the interpretation of the function of genetic variants associated with human diseases and traits.

## Supplementary Material

Supporting Information
