# Supplementary material for: GABPα Binding to Overlapping ETS and CRE DNA Motifs Is Enhanced by CREB1: Custom DNA Microarrays
Source: G3 (Bethesda). 2015 Jul 16;5(9):1909–18. doi: 10.1534/g3.115.020248 (PMC4555227; doi:10.1534/g3.115.020248)
Supplement: Supporting Information [file supp_5_9_1909__index.html]

GABPα Binding to Overlapping ETS and CRE DNA Motifs Is Enhanced by CREB1: Custom DNA Microarrays — Supporting Information 

# GABPα Binding to Overlapping ETS and CRE DNA Motifs Is Enhanced by CREB1: Custom DNA Microarrays

## Supporting Information for He *et al.*, 2015

**Files in this Data Supplement:**

- Supporting Information - Figures S1-S8 and Tables S1-S2 (PDF, 2 MB)
- Figure S1 - Replicates of arrays for each concentration. (PDF, 686 KB)
- Figure S2 - Western blots showing IVT mixtures for GABPα-GST, GABPα-GST+ CREB and CREB. (PDF, 290 KB)
- Figure S3 - CREB1 enhancement of GABPα-GST binding. (PDF, 502 KB)
- Figure S4 - The increase in cooperativity is not due to simple decrease in affinity of the monomer sites. (PDF, 339 KB)
- Figure S5 - CREB1 enhances GABPα binding to several SNPs in the ETS⇔CRE motif (2.5ng concentration). (PDF, 520 KB)
- Figure S6 - CREB1 enhances GABPα binding to several SNPs in the ETS⇔CRE motif (30ng concentration). (PDF, 513 KB)
- Figure S7 - Comparison between universal and custom PBMs. (PDF, 449 KB)
- Figure S8 - GABPα and CREB1 binding to SNPs in genomic regions co-bound by CREB1 and GABPα, in different cell lines. (PDF, 442 KB)
- Table S1 - GABPα peaks with/without CREB1 peaks in 5 cell-lines. (PDF, 115 KB)
- Table S2 - (A) GABPα binding to the consensus and 1bp SNPs of ETS motif in A549 (All Peaks). (B) GABPα binding to the consensus and 1bp SNPs of ETS motif in K562 (All Peaks). (C) GABPα binding to the consensus and 1bp SNPs of ETS motif in HepG2. (D) GABPα binding to the consensus and 1bp SNPs of ETS motif in H1hESC. (E) GABPα binding to the consensus and 1bp SNPs of ETS motif in GM12878. (PDF, 149 KB)
