# Supplementary material for: GABPα Binding to Overlapping ETS and CRE DNA Motifs Is Enhanced by CREB1: Custom DNA Microarrays
Source: G3 (Bethesda). 2015 Jul 16;5(9):1909–18. doi: 10.1534/g3.115.020248 (PMC4555227; doi:10.1534/g3.115.020248)
Supplement: Supporting Information [file supp_g3.115.020248_020248SI.pdf]

G3 · Genes | Genomes | Genetics

GABPα binding to overlapping ETS and CRE DNA motifs is enhanced by CREB1: custom DNA microarrays

Ximiao He*, Khund Sayeed Syed *, Desiree Tillo*, Ishminder Mann*, Matthew T. Weirauch[$@], Charles Vinson*[@]

*Laboratory of Metabolism, NCI, NIH, Bldg. 37, Rm. 3128, Bethesda, MD 20892. [$]Center for Autoimmune Genomics and Etiology, Division of Biomedical Informatics and Division of Developmental Biology, Department of Pediatrics, Cincinnati Children's Hospital Medical Center, University of Cincinnati College of Medicine, Cincinnati, OH 45229.

[@] Co-corresponding authors; MTW: Cincinnati Children's Hospital, 3333 Burnet Avenue, Cincinnati, OH 45229-3039. Tel: (513)-803-9078, Fax: (513)-803-5246, Email: matthew.weirauch@cchmc.org; CV: Bldg. 37, Rm. 3128, Bethesda, MD 20892. Tel: (301) 496-8753, Fax: (301) 496-8419, E-mail: vinsonc@mail.nih.gov

DOI: 10.1534/g3.115.020248

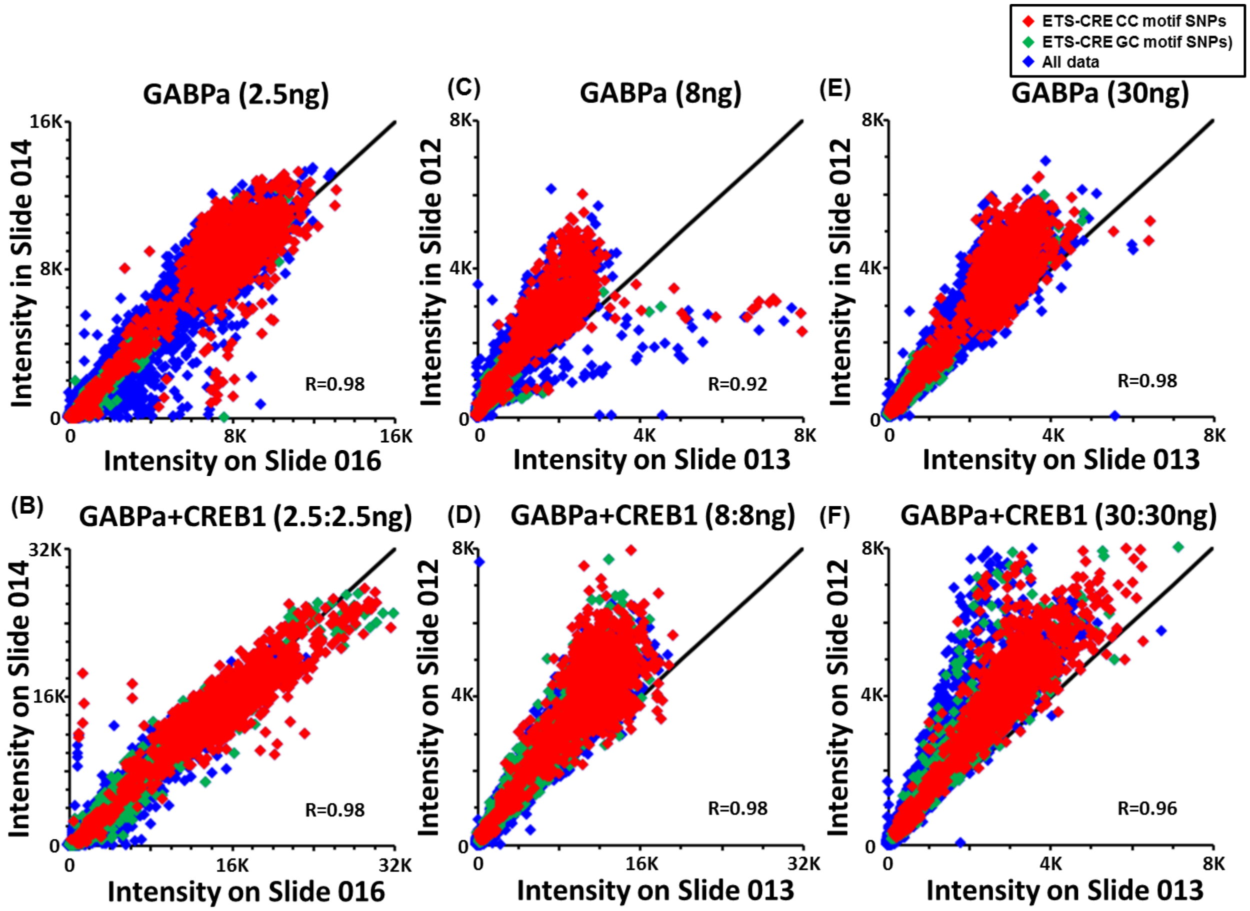

Figure S1 Replicates of arrays for each concentration. Scatter plot depicting fluorescence intensities of each probe in array replicates for different GABPα concentration and GABPα:CREB1 concentration: (A) 2.5ng of GABPα-GST; (B) 2.5ng GABPα-GST plus 2.5ng CREB1; (C) 8ng of GABPα-GST; (D) 8ng GABPα-GST plus 8ng CREB1; (E) 30ng of GABPα-GST; (F) 30ng GABPα-GST plus 30ng CREB1.

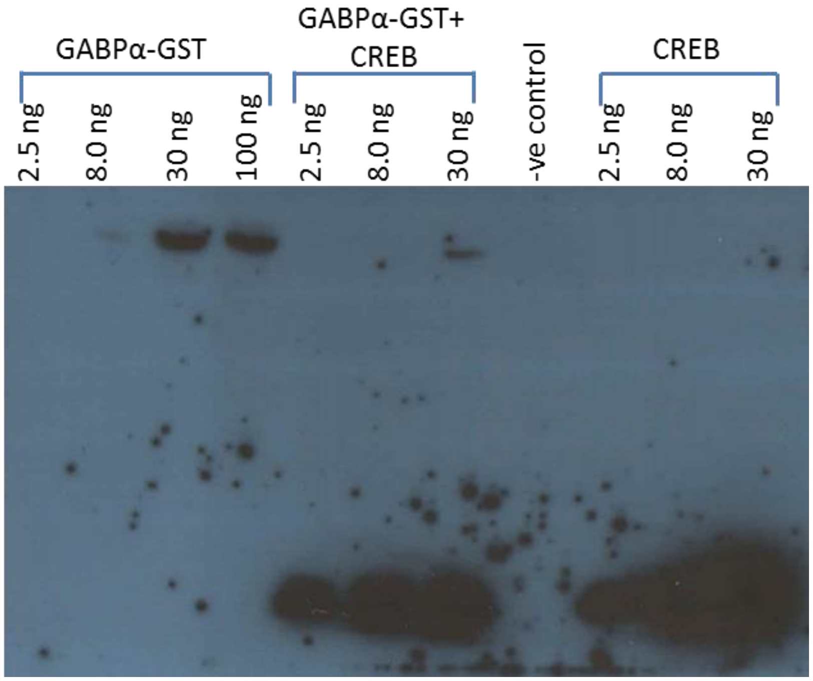

Figure S2 Western blots showing IVT mixtures for GABPα-GST, GABPα-GST+ CREB and CREB. Lanes 1-4 contain increasing amounts of GABPα-GST plasmid (2.5 ng, 8 ng, 30 ng and 100 ng), lanes 5-7 contain plasmid mixtures of GABPα-GST+ CREB in 1:1 ratio (2.5 ng, 8 ng and 30 ng), lane 8 is a negative control (IVT solution without plasmids), and lanes 9-11 show westerns of CREB (2.5 ng, 8 ng and 30 ng). GABPα-GST was detected using anti-GST HRP conjugate and CREB was detected using anti-T7 HRP conjugate.

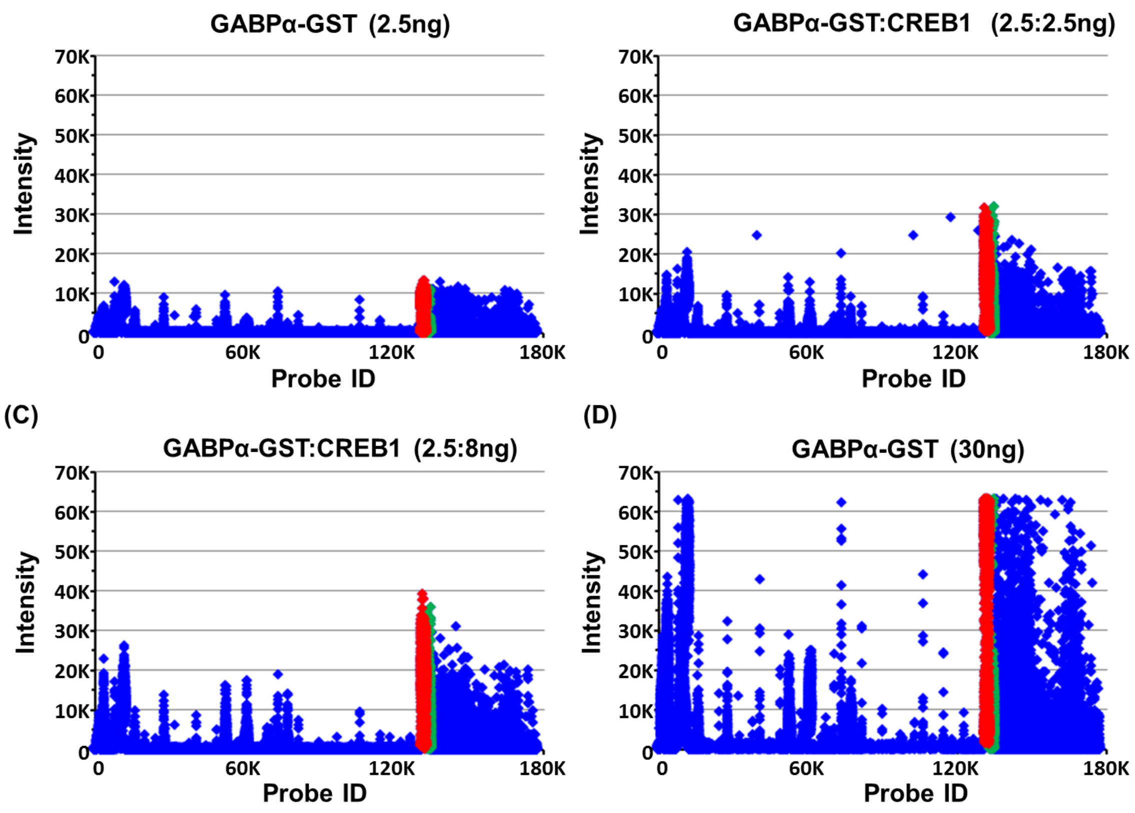

Figure S3 CREB1 enhancement of GABPα-GST binding. Fluorescence intensities of (A) 2.5 ng of GABPα-GST, (B) 2.5 ng GABPα-GST plus 2.5ng CREB1, (C) 2.5ng GABPα-GST plus 8ng CREB1, and (D) 30ng GABPα-GST binding to 177,440 features on the ETS-CRE array. Array probes are ordered as in Figure 1A.

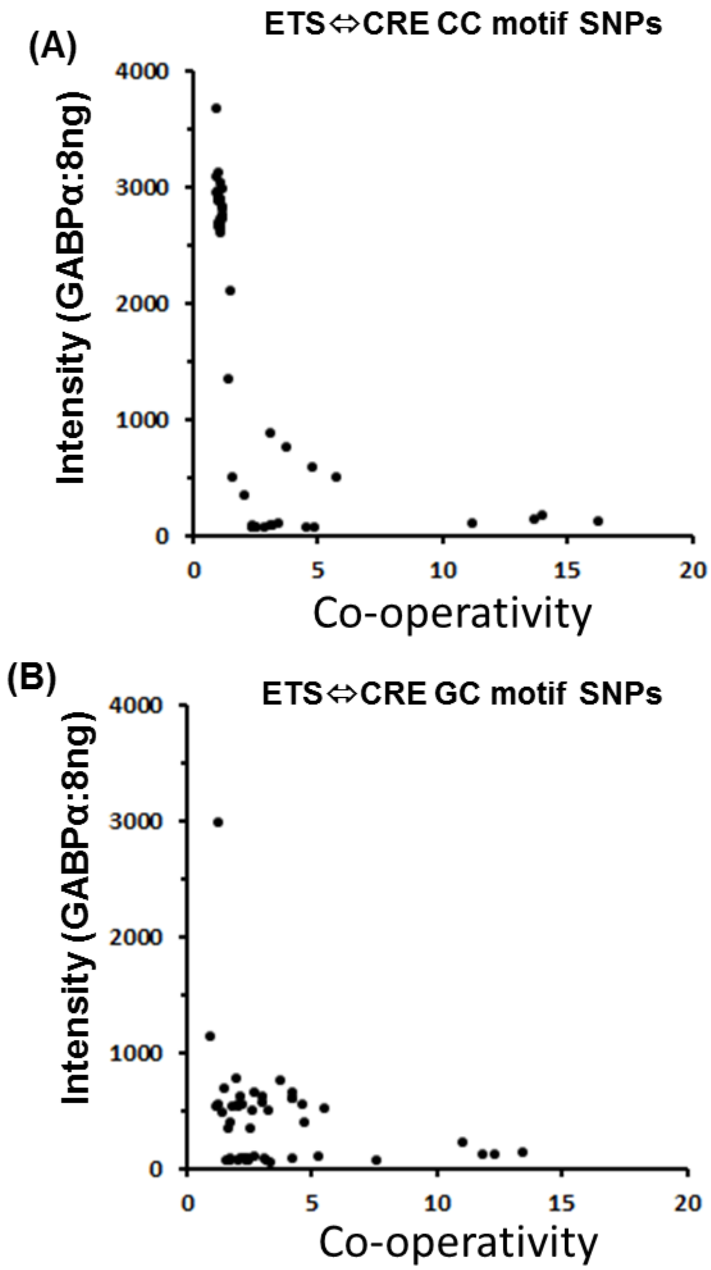

Figure S4 The increase in cooperativity is not due to simple decrease in affinity of the monomer sites. (A-B) Plot of fluorescence intensities of 8 ng of GABPα-GST versus cooperativity with CREB1 measured by ratio of intensity of GABPa-GST binding with/without CREB1 for (A) ETS⇔CRE CC motif SNPs, and (B) ETS⇔CRE CC motif SNPs.

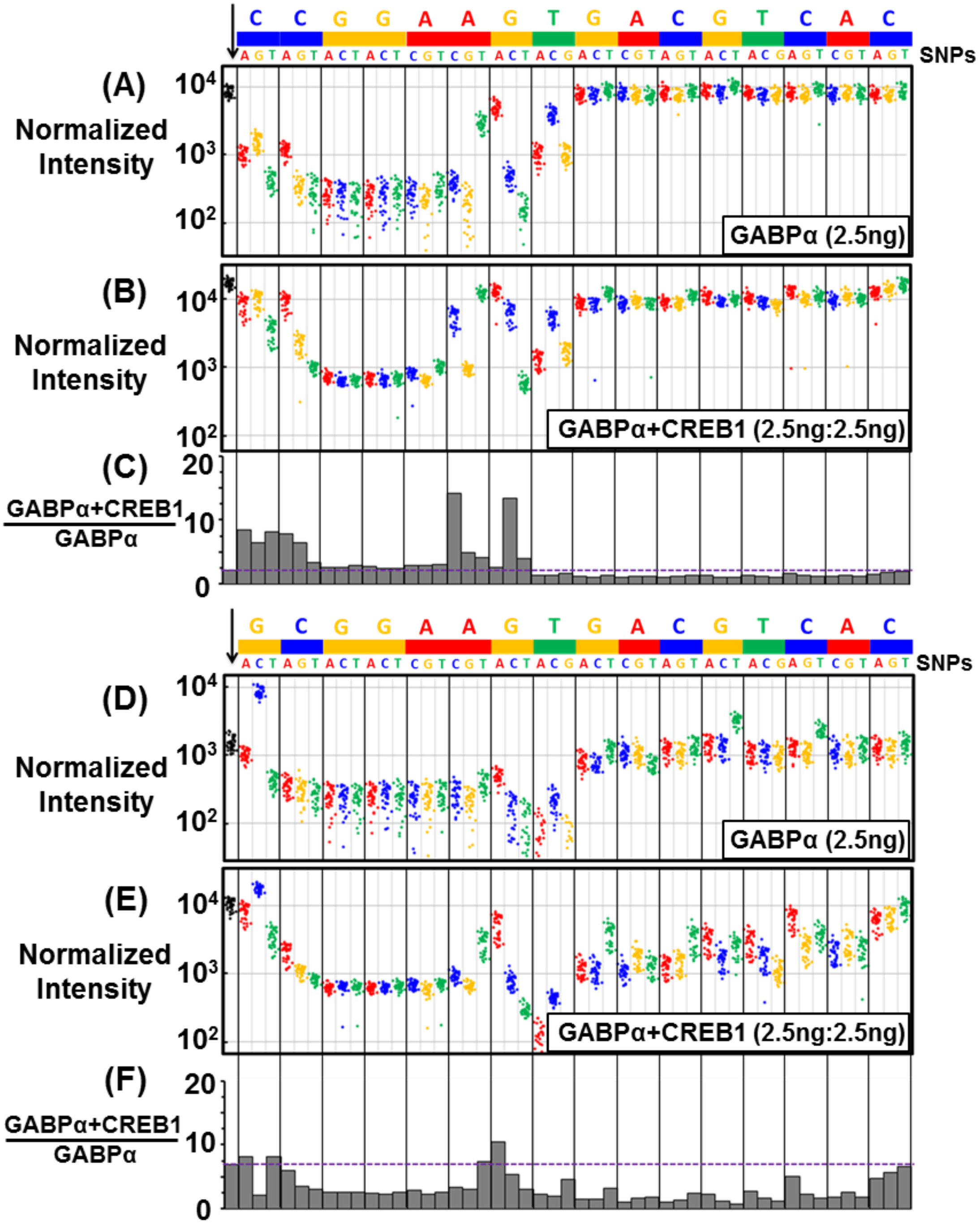

Figure S5 CREB1 enhances GABPα binding to several SNPs in the ETS⇔CRE motif (2.5ng concentration). (A) GABPα-GST (2.5ng) binding to 1,960 features containing the ETS⇔CRE 16-mer CCGGAAGTGACGTCAC and 48 SNPs on the ETS-CRE array. The first column of the figure contains 40 black spots representing GABPα-GST binding to the 40 features containing the consensus ETS⇔CRE motif CCGGAAGTGACGTCAC. The rest of the columns represent 40 features for each of the 48 SNPs, as indicated. (B) GABPα-GST binding in the presence of equal concentration (2.5ng:2.5ng) of the CREB1 plasmid on the ETS-CRE array. (C) Histogram of the ratio of GABPα-GST array intensities +/- CREB1 to the consensus and SNP probes. Horizontal dashed line indicates the ratio of GABPα+CREB1/GABPα binding to the consensus. (D-F) Same as in A-C, but for GABPα-GST (+/- CREB1) binding to 1,960 features containing the weaker ETS⇔CRE 16-mer GCGGAAGTGACGTCAC motif and 48 SNPs on the ETS-CRE array.

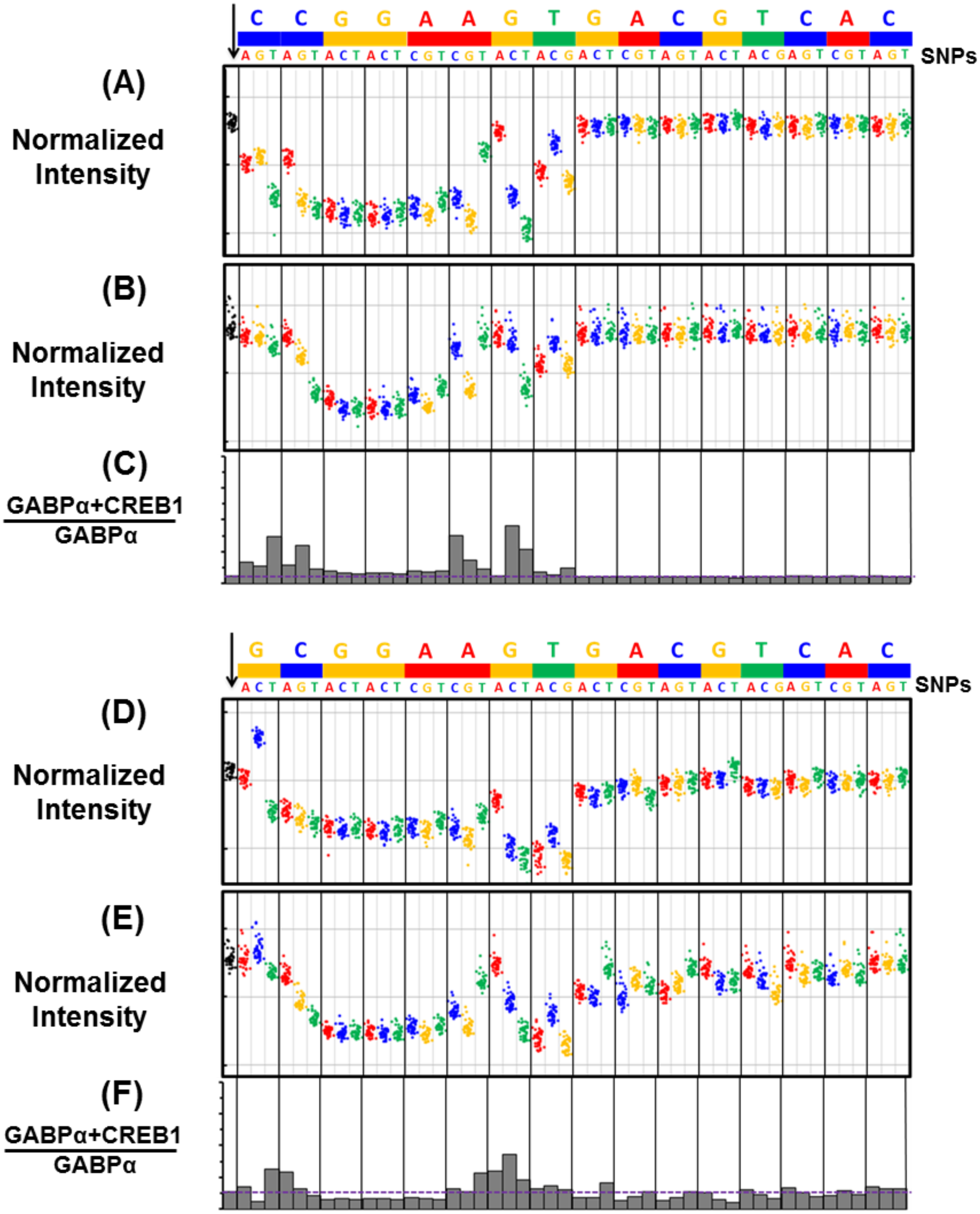

Figure S6 CREB1 enhances GABPα binding to several SNPs in the ETS⇔CRE motif (30ng concentration). Same as in Figures 3 and S5, but using intensities obtained from ETS-CRE PBM experiments using 30ng concentrations of GABPα and CREB1.

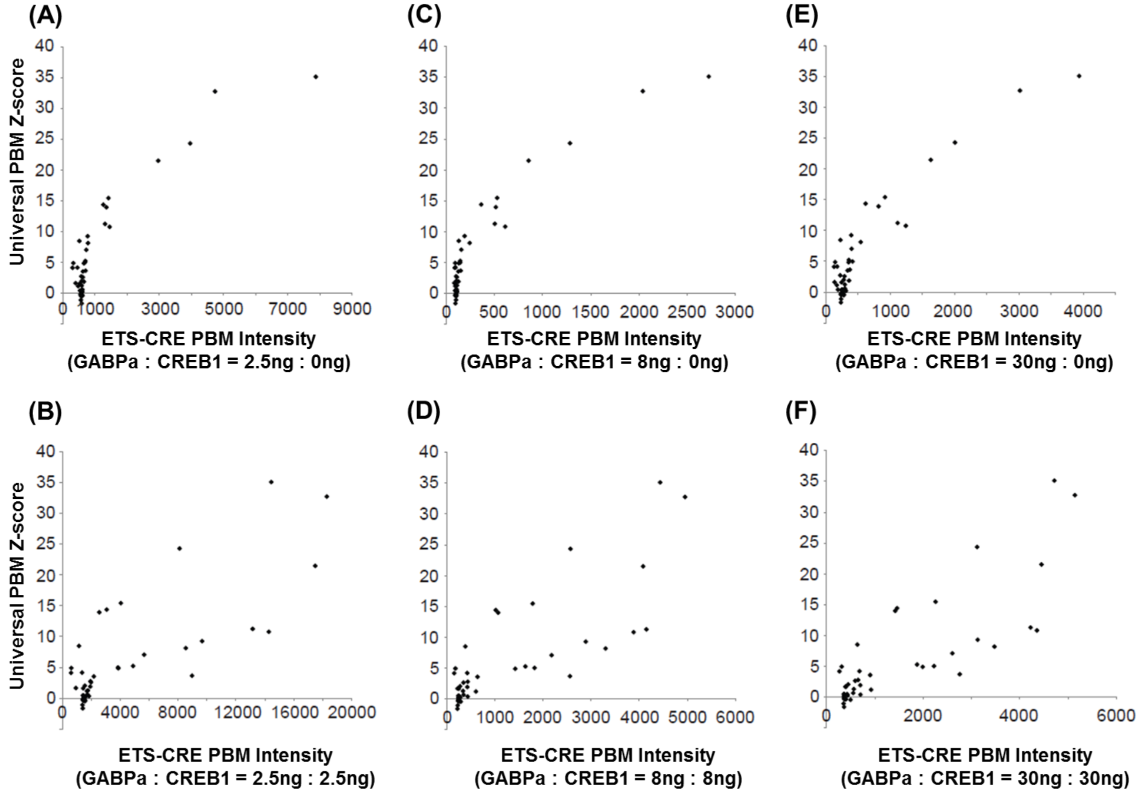

Figure S7 Comparison between universal and custom PBMs. Comparison of custom ETS-CRE array median signal intensity (X axis) and universal PBM Z-scores (Y axis) for each of 46 possible 8 base variants of the ETS motif. (A,C,E) Results using ETS-CRE arrays in the absence of CREB1. (A) 2.5ng GABPα, 0ng CREB1. (C) 8ng GABPα, 0ng CREB1. (E) 30ng GABPα, 0ng CREB1. (B,D,F) Results using ETS-CRE arrays in the presence of equal amounts of CREB1. (B) 2.5ng GABPα, 2.5ng CREB1. (D) 8ng GABPα, 8ng CREB1. (E) 30ng GABPα, 30ng CREB1.

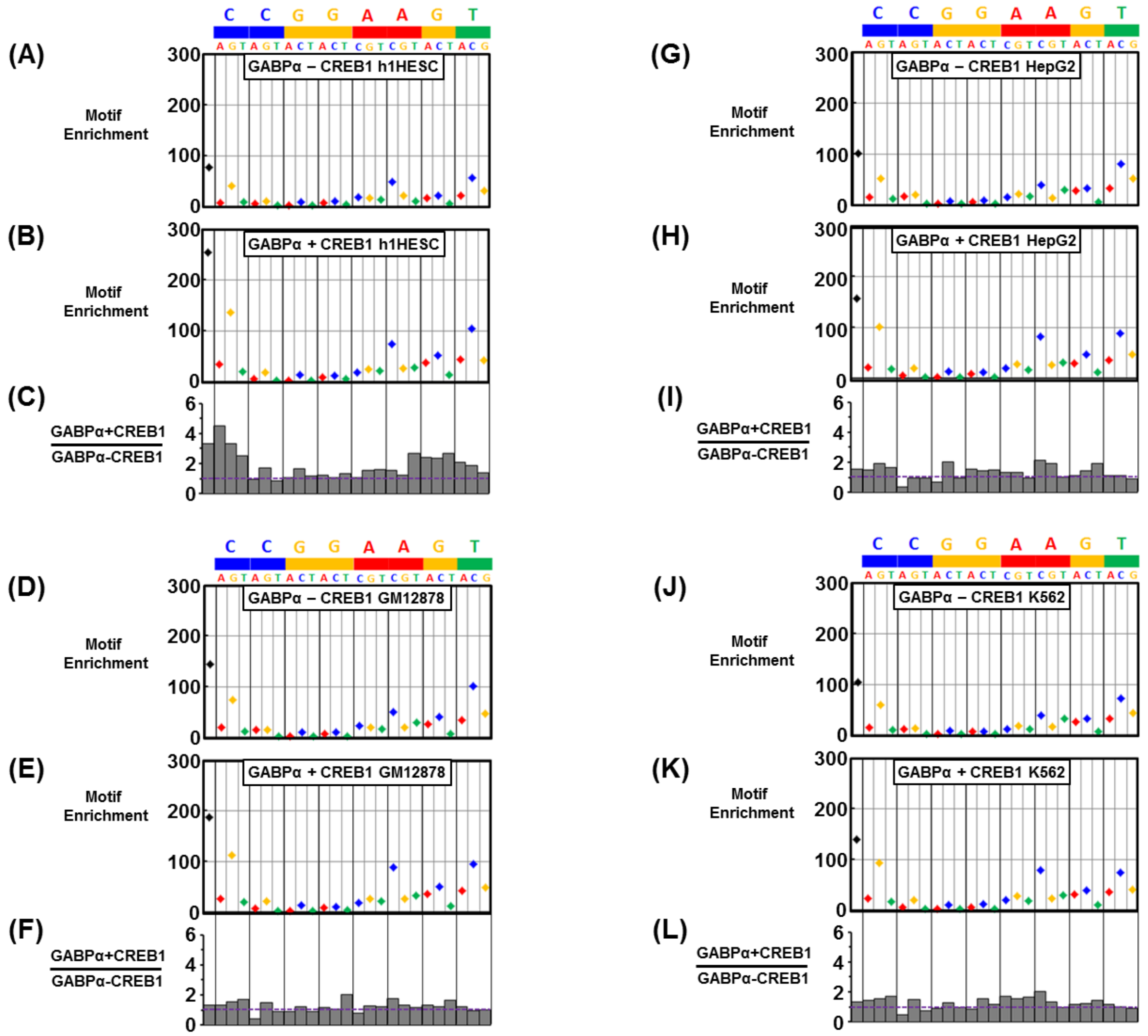

Figure S8 GABPα and CREB1 binding to SNPs in genomic regions co-bound by CREB1 and GABPα, in different cell lines. (A-B) Enrichment of consensus and each 1-bp variation of ETS motif (CCGGAAGT) in GABPα ChIP-seq peaks (A) that do not overlap with CREB1 ChIP-seq peaks (GABPα - CREB1), and (B) that do overlap with CREB1 ChIP-seq peaks (GABPα + CREB1), in h1HESC cells. (C) Histogram of the ratio of motif enrichment +/- CREB1 to the consensus and 1-bp variations of the ETS motif (CCGGAAGT) in h1HESC cells. (D-L) same as in A-C, but for: (D-F) GM12878 cells, (G-I) HepG2 cells, and (J-L) K562 cells.

Table S1 GABPα peaks with/without CREB1 peaks in 5 cell-lines

Cell-lines	All GABPα peaks			GAPBα + CREB1			GABPα – CREB1		
	#	Length	%Genome	#	Length	%Genome	#	Length	%Genome
A549	10,940	5,687,585	0.20%	5,776	1,443,503	0.05%	5,164	4,244,082	0.15%
GM12878	6,381	2,914,811	0.10%	5,298	2,001,359	0.07%	1,083	913,452	0.03%
H1hESC	5,596	2,454,341	0.09%	4,324	1,210,375	0.04%	1,272	1,243,966	0.04%
HepG2	9,776	4,137,716	0.14%	8,873	2,677,258	0.09%	903	1,460,458	0.05%
K562	14,112	5,299,216	0.19%	7,598	1,993,592	0.07%	6,514	3,305,624	0.12%

Table S2A GABPα binding to the consensus and 1bp SNPs of ETS motif in A549 (All Peaks)

ID	Base	Mer	#Genome	GABPα-CREB			GABPα+CREB			Fold increase	p-Value
				#	%	Enrich.	#	%	Enrich.		
0	N	CCGGAAGT	8,608	596	6.9%	46.68	1049	12.2%	241.56	5.17	2.41E-280
1	A	ACGGAAGT	9,033	133	1.5%	9.93	129	1.4%	28.31	2.85	7.10E-19
2	G	GCGGAAGT	6,579	241	3.7%	24.70	468	7.1%	141.01	5.71	2.07E-136
3	T	TCGGAAGT	6,562	64	1.0%	6.58	76	1.2%	22.96	3.49	3.87E-15
4	A	CAGGAAGT	88,465	1,106	1.3%	8.43	300	0.3%	6.72	0.80	4.95E-04
5	G	CGGGAAGT	9,785	153	1.6%	10.54	105	1.1%	21.27	2.02	1.57E-08
6	T	CTGGAAGT	73,134	253	0.3%	2.33	71	0.1%	1.92	0.83	1.52E-01
7	A	CCAGAAGT	67,739	228	0.3%	2.27	58	0.1%	1.70	0.75	4.75E-02
8	C	CCCGAAGT	7,646	73	1.0%	6.44	34	0.4%	8.81	1.37	1.28E-01
9	T	CCTGAAGT	64,674	179	0.3%	1.87	49	0.1%	1.50	0.80	1.77E-01
10	A	CCGAAAGT	6,499	45	0.7%	4.67	23	0.4%	7.02	1.50	1.10E-01
11	C	CCGCAAGT	5,106	41	0.8%	5.41	25	0.5%	9.71	1.79	1.96E-02
12	T	CCGTAAGT	7,499	32	0.4%	2.88	13	0.2%	3.44	1.19	5.89E-01
13	C	CCGGCAGT	7,894	104	1.3%	8.88	74	0.9%	18.58	2.09	6.89E-07
14	G	CCGGGAGT	9,408	166	1.8%	11.90	127	1.3%	26.76	2.25	1.59E-12
15	T	CCGGTAGT	2,777	40	1.4%	9.71	25	0.9%	17.84	1.84	1.54E-02
16	C	CCGGACGT	1,756	49	2.8%	18.81	81	4.6%	91.44	4.86	3.85E-22
17	G	CCGGAGGT	8,121	149	1.8%	12.37	92	1.1%	22.46	1.82	5.02E-06
18	T	CCGGATGT	6,008	132	2.2%	14.81	112	1.9%	36.95	2.49	1.76E-13
19	A	CCGGAAAT	7,627	174	2.3%	15.38	175	2.3%	45.48	2.96	2.15E-26
20	C	CCGGAACT	6,036	151	2.5%	16.87	168	2.8%	55.17	3.27	4.17E-29
21	T	CCGGAATT	5,550	53	1.0%	6.44	28	0.5%	10.00	1.55	5.74E-02
22	A	CCGGAAGA	10,630	249	2.3%	15.79	235	2.2%	43.82	2.77	1.07E-31
23	C	CCGGAAGC	11,970	509	4.3%	28.67	738	6.2%	122.21	4.26	1.25E-165
24	G	CCGGAAGG	12,065	361	3.0%	20.17	312	2.6%	51.26	2.54	6.88E-36

"Enrich." is the enrichment score for each motif (see also Method). P-Value is calculated based on Fisher's exact test.

Table S2B GABPα binding to the consensus and 1bp SNPs of ETS motif in K562 (All Peaks)

ID	Base	Mer	#Genome	GABPα-CREB			GABPα+CREB			Fold increase	p-Value
				#	%	Enrich.	#	%	Enrich.		
0	N	CCGGAAGT	8,608	1,043	12.1%	104.88	835	9.7%	139.23	1.33	9.34E-10
1	A	ACGGAAGT	9,033	161	1.8%	15.43	139	1.5%	22.09	1.43	1.84E-03
2	G	GCGGAAGT	6,579	451	6.9%	59.34	424	6.4%	92.50	1.56	3.66E-11
3	T	TCGGAAGT	6,562	73	1.1%	9.63	75	1.1%	16.40	1.70	1.04E-03
4	A	CAGGAAGT	88,465	1,211	1.4%	11.85	335	0.4%	5.44	0.46	2.43E-38
5	G	CGGGAAGT	9,785	146	1.5%	12.92	132	1.3%	19.36	1.50	6.88E-04
6	T	CTGGAAGT	73,134	191	0.3%	2.26	84	0.1%	1.65	0.73	1.54E-02
7	A	CCAGAAGT	67,739	158	0.2%	2.02	84	0.1%	1.78	0.88	3.50E-01
8	C	CCCGAAGT	7,646	70	0.9%	7.92	53	0.7%	9.95	1.26	2.11E-01
9	T	CCTGAAGT	64,674	132	0.2%	1.77	74	0.1%	1.64	0.93	6.15E-01
10	A	CCGAAAGT	6,499	46	0.7%	6.13	24	0.4%	5.30	0.87	5.65E-01
11	C	CCGCAAGT	5,106	44	0.9%	7.46	41	0.8%	11.52	1.55	4.34E-02
12	T	CCGTAAGT	7,499	20	0.3%	2.31	14	0.2%	2.68	1.16	6.69E-01
13	C	CCGGCAGT	7,894	103	1.3%	11.29	106	1.3%	19.27	1.71	9.28E-05
14	G	CCGGGAGT	9,408	190	2.0%	17.48	180	1.9%	27.46	1.57	1.19E-05
15	T	CCGGTAGT	2,777	36	1.3%	11.22	36	1.3%	18.61	1.66	3.01E-02
16	C	CCGGACGT	1,756	79	4.5%	38.94	96	5.5%	78.47	2.01	2.51E-06
17	G	CCGGAGGT	8,121	159	2.0%	16.95	128	1.6%	22.62	1.33	1.47E-02
18	T	CCGGATGT	6,008	220	3.7%	31.70	123	2.0%	29.38	0.93	5.01E-01
19	A	CCGGAAAT	7,627	233	3.1%	26.44	161	2.1%	30.30	1.15	1.84E-01
20	C	CCGGAACT	6,036	221	3.7%	31.69	163	2.7%	38.76	1.22	5.08E-02
21	T	CCGGAATT	5,550	43	0.8%	6.71	37	0.7%	9.57	1.43	1.11E-01
22	A	CCGGAAGA	10,630	387	3.6%	31.51	268	2.5%	36.19	1.15	8.17E-02
23	C	CCGGAAGC	11,970	1,000	8.4%	72.31	611	5.1%	73.26	1.01	8.00E-01
24	G	CCGGAAGG	12,065	607	5.0%	43.55	337	2.8%	40.09	0.92	2.23E-01

"Enrich." is the enrichment score for each motif (see also Method). P-Value is calculated based on Fisher's exact test.

Table S2C GABPα binding to the consensus and 1bp SNPs of ETS motif in HepG2

ID	Base	Mer	#Genome	GABPα-CREB			GABPα+CREB			Fold increase	p-Value
				#	%	Enrich.	#	%	Enrich.		
0	N	CCGGAAGT	8,608	445	5.2%	101.28	1262	14.7%	156.69	1.55	1.50E-15
1	A	ACGGAAGT	9,033	66	0.7%	14.32	182	2.0%	21.53	1.50	4.22E-03
2	G	GCGGAAGT	6,579	174	2.6%	51.82	618	9.4%	100.39	1.94	4.23E-15
3	T	TCGGAAGT	6,562	37	0.6%	11.05	111	1.7%	18.08	1.64	8.77E-03
4	A	CAGGAAGT	88,465	716	0.8%	15.86	452	0.5%	5.46	0.34	3.37E-77
5	G	CGGGAAGT	9,785	99	1.0%	19.82	174	1.8%	19.00	0.96	7.38E-01
6	T	CTGGAAGT	73,134	72	0.1%	1.93	125	0.2%	1.83	0.95	7.13E-01
7	A	CCAGAAGT	67,739	78	0.1%	2.26	97	0.1%	1.53	0.68	1.02E-02
8	C	CCCGAAGT	7,646	24	0.3%	6.15	89	1.2%	12.44	2.02	1.77E-03
9	T	CCTGAAGT	64,674	49	0.1%	1.48	87	0.1%	1.44	0.97	8.58E-01
10	A	CCGAAAGT	6,499	16	0.2%	4.82	46	0.7%	7.56	1.57	1.18E-01
11	C	CCGCAAGT	5,106	22	0.4%	8.44	57	1.1%	11.93	1.41	1.66E-01
12	T	CCGTAAGT	7,499	7	0.1%	1.83	19	0.3%	2.71	1.48	3.72E-01
13	C	CCGGCAGT	7,894	58	0.7%	14.39	140	1.8%	18.95	1.32	7.71E-02
14	G	CCGGGAGT	9,408	100	1.1%	20.82	247	2.6%	28.06	1.35	1.16E-02
15	T	CCGGTAGT	2,777	24	0.9%	16.93	41	1.5%	15.78	0.93	7.84E-01
16	C	CCGGACGT	1,756	34	1.9%	37.93	133	7.6%	80.95	2.13	5.36E-05
17	G	CCGGAGGT	8,121	56	0.7%	13.51	196	2.4%	25.79	1.91	1.41E-05
18	T	CCGGATGT	6,008	91	1.5%	29.68	171	2.8%	30.42	1.03	8.49E-01
19	A	CCGGAAAT	7,627	104	1.4%	26.72	213	2.8%	29.85	1.12	3.54E-01
20	C	CCGGAACT	6,036	101	1.7%	32.78	263	4.4%	46.57	1.42	2.58E-03
21	T	CCGGAATT	5,550	16	0.3%	5.65	57	1.0%	10.98	1.94	1.68E-02
22	A	CCGGAAGA	10,630	173	1.6%	31.89	360	3.4%	36.20	1.14	1.70E-01
23	C	CCGGAAGC	11,970	485	4.1%	79.38	987	8.2%	88.13	1.11	5.94E-02
24	G	CCGGAAGG	12,065	319	2.6%	51.80	520	4.3%	46.06	0.89	9.86E-02

"Enrich." is the enrichment score for each motif (see also Method). P-Value is calculated based on Fisher's exact test.

Table S2D GABPα binding to the consensus and 1bp SNPs of ETS motif in H1hESC

ID	Base	Mer	#Genome	GABPα-CREB			GABPα+CREB			Fold increase	p-Value
				#	%	Enrich.	#	%	Enrich.		
0	N	CCGGAAGT	8,608	285	3.3%	76.16	926	10.8%	254.31	3.34	1.19E-79
1	A	ACGGAAGT	9,033	29	0.3%	7.38	127	1.4%	33.24	4.50	1.08E-15
2	G	GCGGAAGT	6,579	117	1.8%	40.91	378	5.7%	135.83	3.32	2.28E-33
3	T	TCGGAAGT	6,562	23	0.4%	8.06	56	0.9%	20.17	2.50	1.26E-04
4	A	CAGGAAGT	88,465	197	0.2%	5.12	181	0.2%	4.84	0.94	5.78E-01
5	G	CGGGAAGT	9,785	44	0.4%	10.34	72	0.7%	17.39	1.68	6.01E-03
6	T	CTGGAAGT	73,134	70	0.1%	2.20	58	0.1%	1.87	0.85	3.65E-01
7	A	CCAGAAGT	67,739	53	0.1%	1.80	55	0.1%	1.92	1.07	7.38E-01
8	C	CCCGAAGT	7,646	25	0.3%	7.52	40	0.5%	12.37	1.64	4.87E-02
9	T	CCTGAAGT	64,674	44	0.1%	1.56	49	0.1%	1.79	1.14	5.15E-01
10	A	CCGAAAGT	6,499	18	0.3%	6.37	21	0.3%	7.64	1.20	5.71E-01
11	C	CCGCAAGT	5,106	23	0.5%	10.36	24	0.5%	11.11	1.07	8.11E-01
12	T	CCGTAAGT	7,499	11	0.1%	3.37	14	0.2%	4.41	1.31	5.04E-01
13	C	CCGGCAGT	7,894	59	0.7%	17.19	60	0.8%	17.97	1.05	8.10E-01
14	G	CCGGGAGT	9,408	65	0.7%	15.89	98	1.0%	24.63	1.55	5.78E-03
15	T	CCGGTAGT	2,777	16	0.6%	13.25	25	0.9%	21.28	1.61	1.35E-01
16	C	CCGGACGT	1,756	37	2.1%	48.47	55	3.1%	74.04	1.53	4.46E-02
17	G	CCGGAGGT	8,121	73	0.9%	20.68	88	1.1%	25.62	1.24	1.75E-01
18	T	CCGGATGT	6,008	27	0.4%	10.34	71	1.2%	27.94	2.70	4.64E-06
19	A	CCGGAAAT	7,627	52	0.7%	15.68	121	1.6%	37.50	2.39	5.75E-08
20	C	CCGGAACT	6,036	57	0.9%	21.72	132	2.2%	51.70	2.38	1.66E-08
21	T	CCGGAATT	5,550	12	0.2%	4.97	31	0.6%	13.20	2.66	2.81E-03
22	A	CCGGAAGA	10,630	96	0.9%	20.77	195	1.8%	43.37	2.09	1.57E-09
23	C	CCGGAAGC	11,970	290	2.4%	55.73	531	4.4%	104.87	1.88	1.32E-18
24	G	CCGGAAGG	12,065	158	1.3%	30.12	211	1.7%	41.34	1.37	2.51E-03

"Enrich." is the enrichment score for each motif (see also Method). P-Value is calculated based on Fisher's exact test.

Table S2E GABPα binding to the consensus and 1bp SNPs of ETS motif in GM12878

ID	Base	Mer	#Genome	GABPα-CREB			GABPα+CREB			Fold increase	p-Value
				#	%	Enrich.	#	%	Enrich.		
0	N	CCGGAAGT	8,608	394	4.6%	143.38	1,130	13.1%	187.68	1.31	3.91E-06
1	A	ACGGAAGT	9,033	57	0.6%	19.77	167	1.8%	26.43	1.34	5.73E-02
2	G	GCGGAAGT	6,579	155	2.4%	73.80	519	7.9%	112.79	1.53	3.04E-06
3	T	TCGGAAGT	6,562	24	0.4%	11.46	89	1.4%	19.39	1.69	2.06E-02
4	A	CAGGAAGT	88,465	409	0.5%	14.48	383	0.4%	6.19	0.43	7.27E-35
5	G	CGGGAAGT	9,785	45	0.5%	14.41	146	1.5%	21.33	1.48	2.05E-02
6	T	CTGGAAGT	73,134	43	0.1%	1.84	86	0.1%	1.68	0.91	6.25E-01
7	A	CCAGAAGT	67,739	44	0.1%	2.03	89	0.1%	1.88	0.92	6.65E-01
8	C	CCCGAAGT	7,646	25	0.3%	10.24	68	0.9%	12.72	1.24	3.54E-01
9	T	CCTGAAGT	64,674	37	0.1%	1.79	73	0.1%	1.61	0.90	6.03E-01
10	A	CCGAAAGT	6,499	14	0.2%	6.75	36	0.6%	7.92	1.17	6.11E-01
11	C	CCGCAAGT	5,106	16	0.3%	9.82	37	0.7%	10.36	1.06	8.57E-01
12	T	CCGTAAGT	7,499	4	0.1%	1.67	18	0.2%	3.43	2.05	1.83E-01
13	C	CCGGCAGT	7,894	59	0.7%	23.41	102	1.3%	18.47	0.79	1.47E-01
14	G	CCGGGAGT	9,408	60	0.6%	19.98	171	1.8%	25.99	1.30	7.88E-02
15	T	CCGGTAGT	2,777	15	0.5%	16.92	40	1.4%	20.59	1.22	5.16E-01
16	C	CCGGACGT	1,756	28	1.6%	49.95	109	6.2%	88.75	1.78	5.95E-03
17	G	CCGGAGGT	8,121	51	0.6%	19.67	148	1.8%	26.06	1.32	8.25E-02
18	T	CCGGATGT	6,008	55	0.9%	28.68	138	2.3%	32.84	1.15	3.95E-01
19	A	CCGGAAAT	7,627	64	0.8%	26.29	187	2.5%	35.05	1.33	4.61E-02
20	C	CCGGAACT	6,036	79	1.3%	41.00	207	3.4%	49.03	1.20	1.76E-01
21	T	CCGGAATT	5,550	13	0.2%	7.34	47	0.8%	12.11	1.65	1.06E-01
22	A	CCGGAAGA	10,630	115	1.1%	33.89	307	2.9%	41.29	1.22	7.03E-02
23	C	CCGGAAGC	11,970	387	3.2%	101.27	787	6.6%	94.00	0.93	2.30E-01
24	G	CCGGAAGG	12,065	182	1.5%	47.25	412	3.4%	48.82	1.03	7.14E-01

"Enrich." is the enrichment score for each motif (see also Method). P-Value is calculated based on Fisher's exact test.
