# Supplementary material for: GABPα Binding to Overlapping ETS and CRE DNA Motifs Is Enhanced by CREB1: Custom DNA Microarrays
Source: G3 (Bethesda). 2015 Jul 16;5(9):1909–18. doi: 10.1534/g3.115.020248 (PMC4555227; doi:10.1534/g3.115.020248)
Supplement: Supporting Information [file supp_g3.115.020248_FigureS1.pdf]

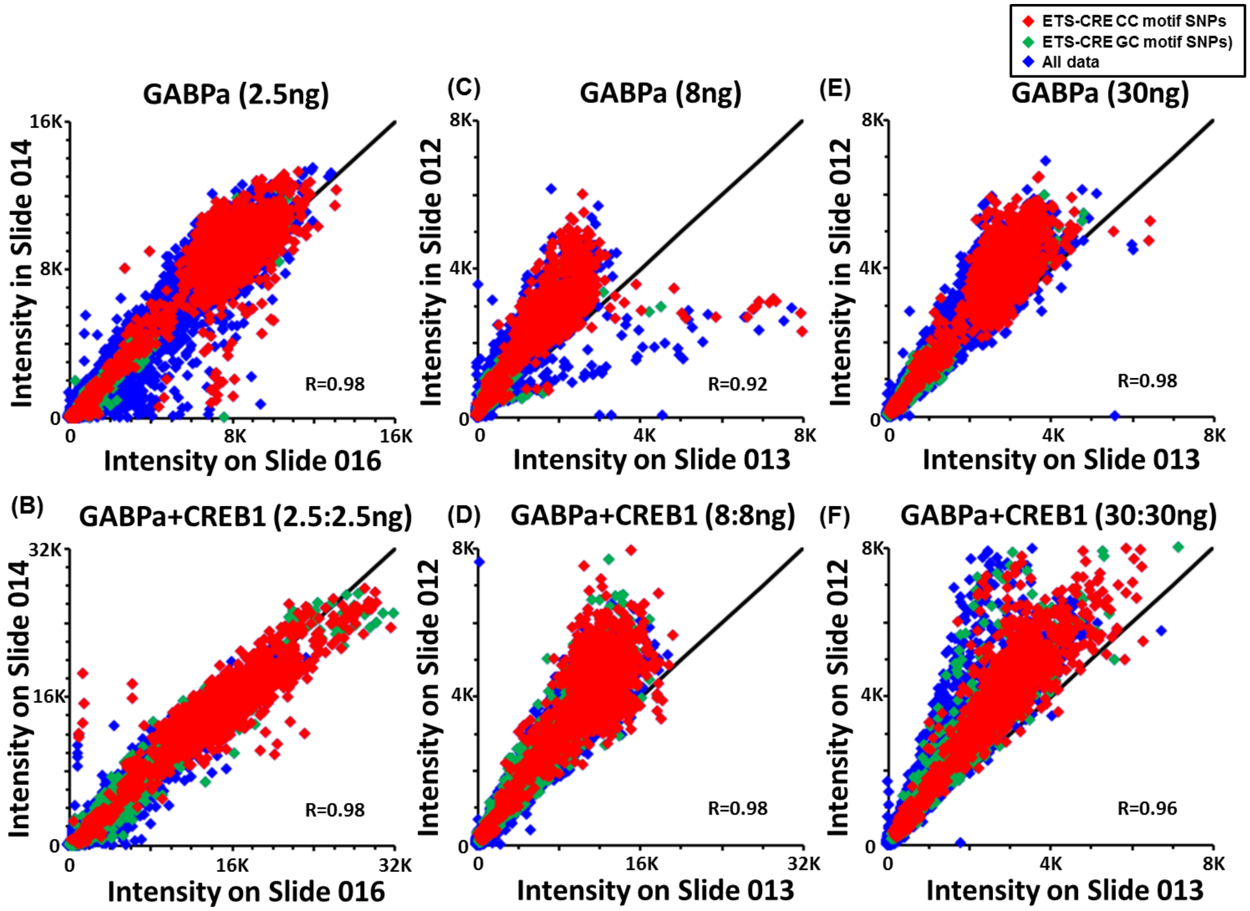

Figure S1 Replicates of arrays for each concentration. Scatter plot depicting fluorescence intensities of each probe in array replicates for different GABPα concentration and GABPα:CREB1 concentration: (A) 2.5ng of GABPα-GST; (B) 2.5ng GABPα-GST plus 2.5ng CREB1; (C) 8ng of GABPα-GST; (D) 8ng GABPα-GST plus 8ng CREB1; (E) 30ng of GABPα-GST; (F) 30ng GABPα-GST plus 30ng CREB1.
