# Supplementary material for: GABPα Binding to Overlapping ETS and CRE DNA Motifs Is Enhanced by CREB1: Custom DNA Microarrays
Source: G3 (Bethesda). 2015 Jul 16;5(9):1909–18. doi: 10.1534/g3.115.020248 (PMC4555227; doi:10.1534/g3.115.020248)
Supplement: Supporting Information [file supp_g3.115.020248_FigureS2.pdf]

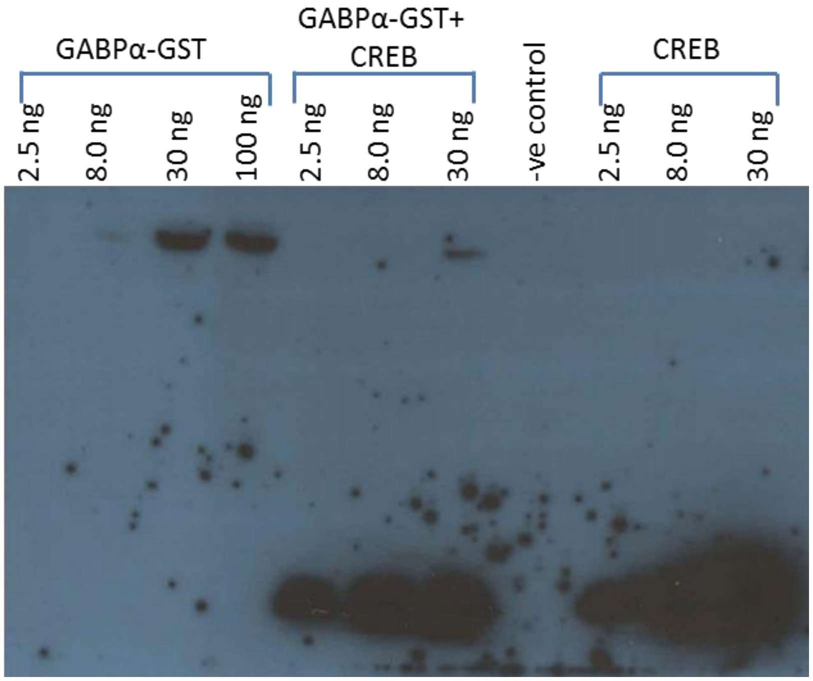


**Figure S2 Western blots showing IVT mixtures for GABPα-GST, GABPα-GST+ CREB and CREB.** Lanes 1-4 contain increasing amounts of GABPα-GST plasmid (2.5 ng, 8 ng, 30 ng and 100 ng), lanes 5-7 contain plasmid mixtures of GABPα-GST+ CREB in 1:1 ratio (2.5 ng, 8 ng and 30 ng ), lane 8 is a negative control (IVT solution without plasmids), and lanes 9-11 show westerns of CREB (2.5 ng, 8 ng and 30 ng ). GABPα-GST was detected using anti-GST HRP conjugate and CREB was detected using anti-T7 HRP conjugate.
