# Supplementary material for: GABPα Binding to Overlapping ETS and CRE DNA Motifs Is Enhanced by CREB1: Custom DNA Microarrays
Source: G3 (Bethesda). 2015 Jul 16;5(9):1909–18. doi: 10.1534/g3.115.020248 (PMC4555227; doi:10.1534/g3.115.020248)
Supplement: Supporting Information [file supp_g3.115.020248_FigureS3.pdf]

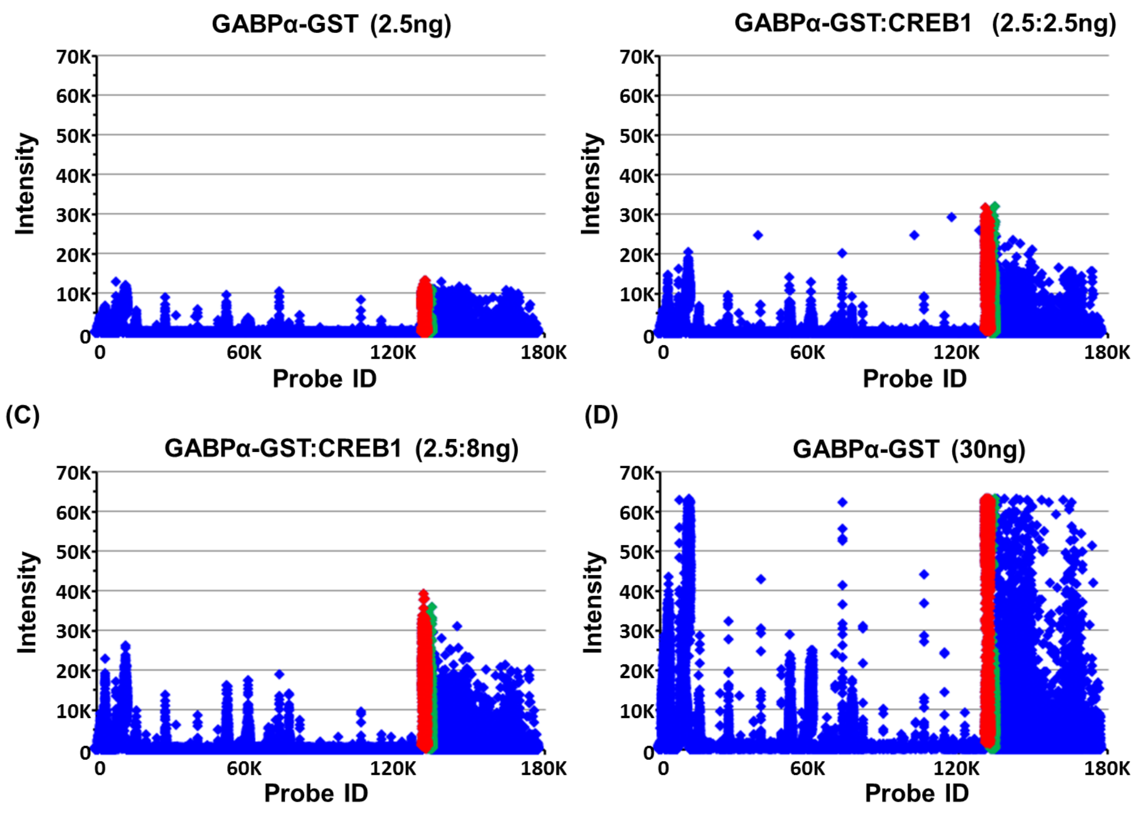

Figure S3 CREB1 enhancement of GABPα-GST binding. Fluorescence intensities of (A) 2.5 ng of GABPα-GST, (B) 2.5 ng GABPα-GST plus 2.5ng CREB1, (C) 2.5ng GABPα-GST plus 8ng CREB1, and (D) 30ng GABPα-GST binding to 177,440 features on the ETS-CRE array. Array probes are ordered as in Figure 1A.
