# Supplementary material for: GABPα Binding to Overlapping ETS and CRE DNA Motifs Is Enhanced by CREB1: Custom DNA Microarrays
Source: G3 (Bethesda). 2015 Jul 16;5(9):1909–18. doi: 10.1534/g3.115.020248 (PMC4555227; doi:10.1534/g3.115.020248)
Supplement: Supporting Information [file supp_g3.115.020248_FigureS4.pdf]

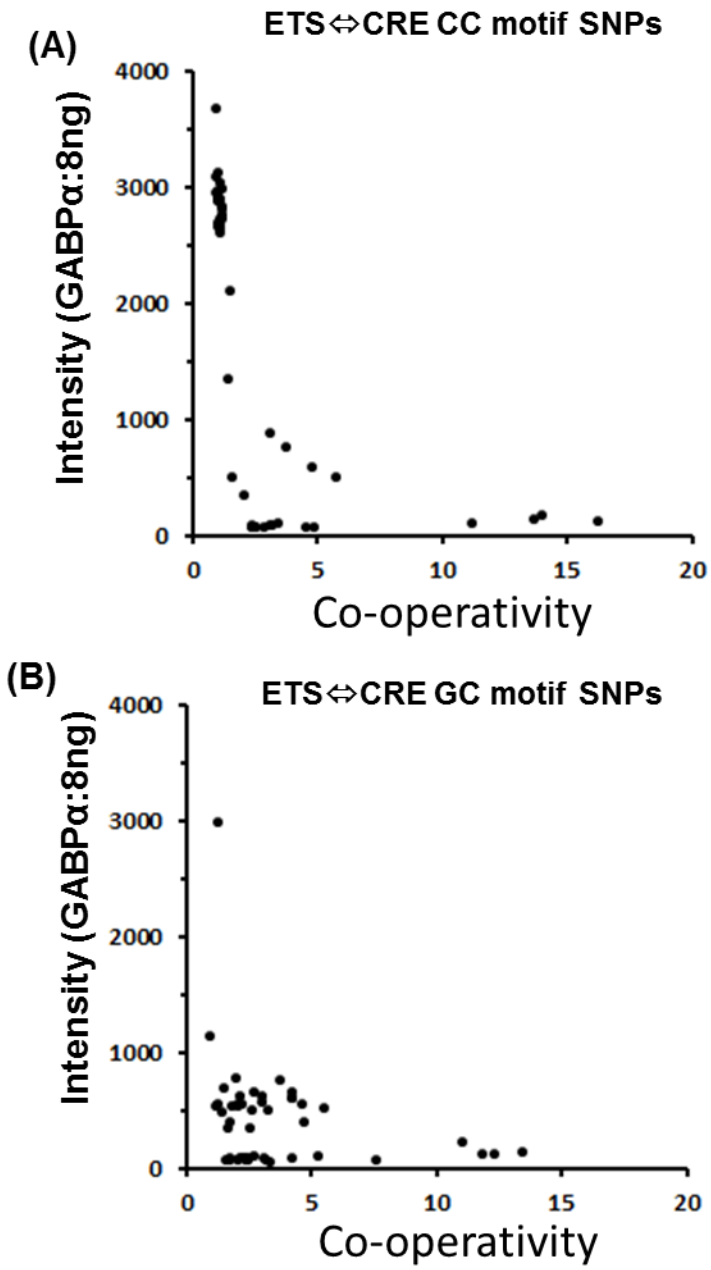

Figure S4 The increase in cooperativity is not due to simple decrease in affinity of the monomer sites. (A-B) Plot of fluorescence intensities of 8 ng of GABPα-GST versus cooperativity with CREB1 measured by ratio of intensity of GABPa-GST binding with/without CREB1 for (A) ETS⇔CRE CC motif SNPs, and (B) ETS⇔CRE CC motif SNPs.
