# Supplementary material for: GABPα Binding to Overlapping ETS and CRE DNA Motifs Is Enhanced by CREB1: Custom DNA Microarrays
Source: G3 (Bethesda). 2015 Jul 16;5(9):1909–18. doi: 10.1534/g3.115.020248 (PMC4555227; doi:10.1534/g3.115.020248)
Supplement: Supporting Information [file supp_g3.115.020248_FigureS5.pdf]

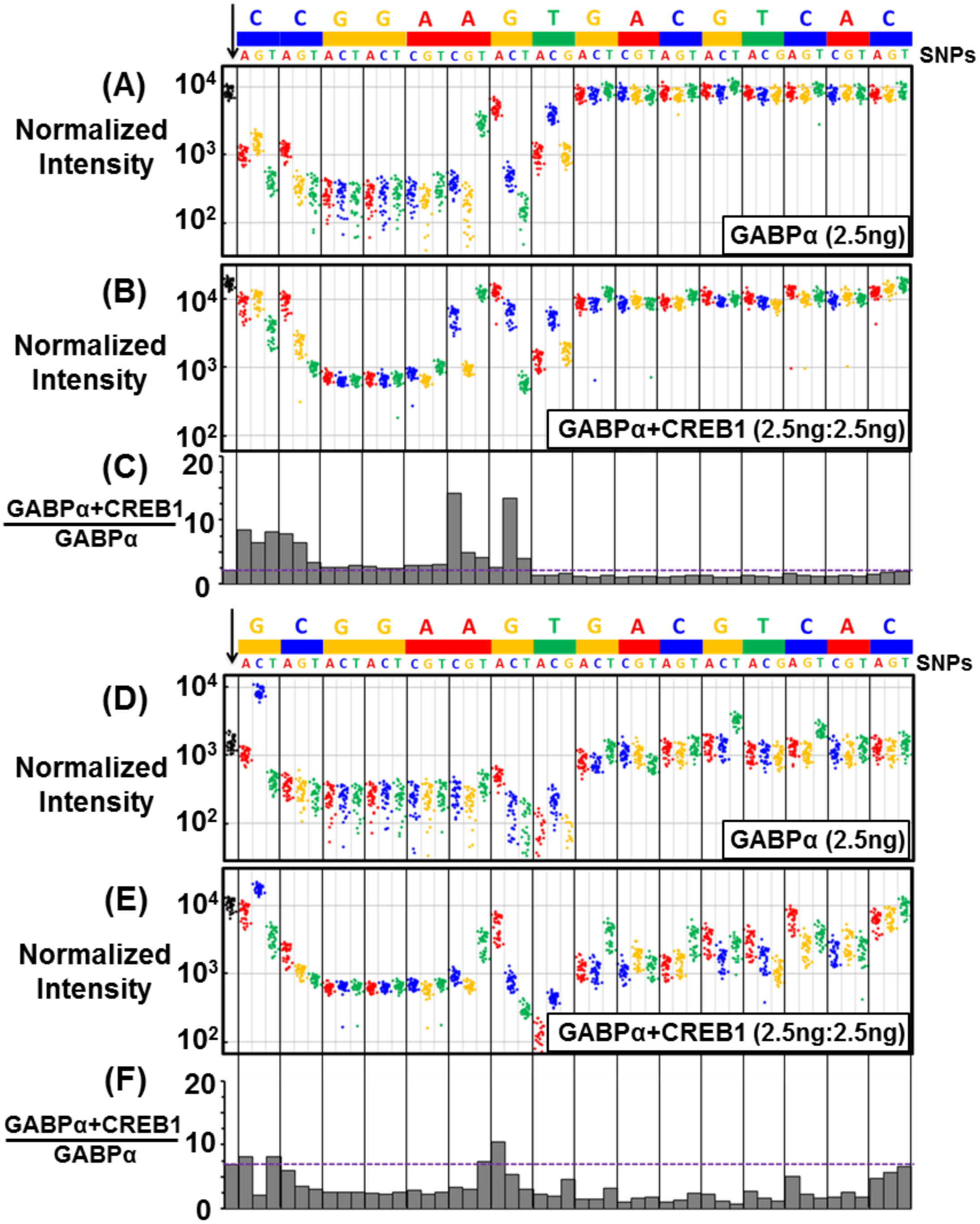

Figure S5 CREB1 enhances GABPα binding to several SNPs in the ETS⬄CRE motif (2.5ng concentration). (A) GABPα-GST (2.5ng) binding to 1,960 features containing the ETS⬄CRE 16-mer CCGGAAGTGACGTCAC and 48 SNPs on the ETS-CRE array. The first column of the figure contains 40 black spots representing GABPα-GST binding to the 40 features containing the consensus ETS⬄CRE motif CCGGAAGTGACGTCAC. The rest of the columns represent 40 features for each of the 48 SNPs, as indicated. (B) GABPα-GST binding in the presence of equal concentration (2.5ng:2.5ng) of the CREB1 plasmid on the ETS-CRE array. (C) Histogram of the ratio of GABPα-GST array intensities +/- CREB1 to the consensus and SNP probes. Horizontal dashed line indicates the ratio of GABPα+CREB1/GABPα binding to the consensus. (D-F) Same as in A-C, but for GABPα-GST (+/- CREB1) binding to 1,960 features containing the weaker ETS⬄CRE 16-mer GCGGAAGTGACGTCAC motif and 48 SNPs on the ETS-CRE array.
