# Supplementary material for: GABPα Binding to Overlapping ETS and CRE DNA Motifs Is Enhanced by CREB1: Custom DNA Microarrays
Source: G3 (Bethesda). 2015 Jul 16;5(9):1909–18. doi: 10.1534/g3.115.020248 (PMC4555227; doi:10.1534/g3.115.020248)
Supplement: Supporting Information [file supp_g3.115.020248_FigureS6.pdf]

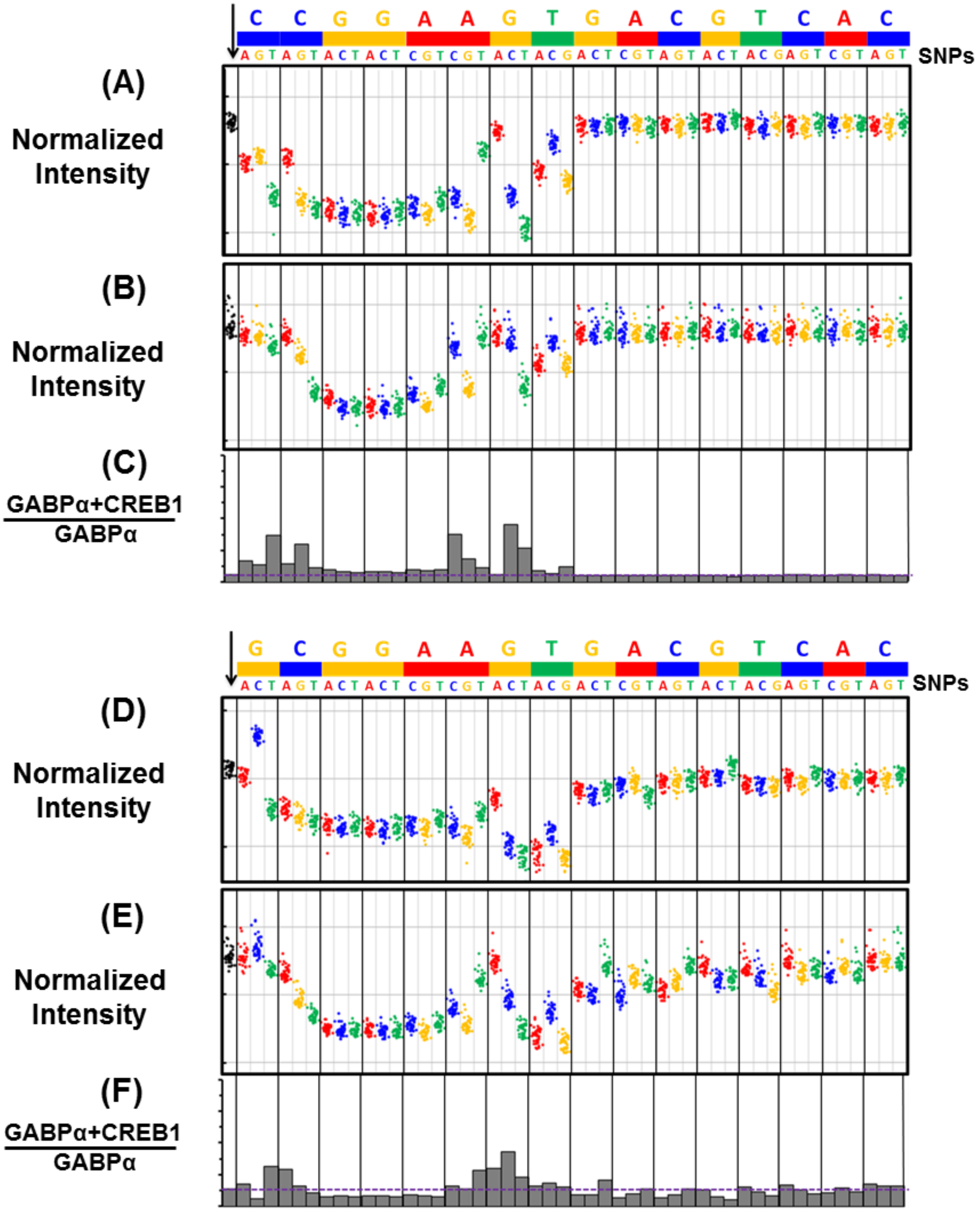

Figure S6 CREB1 enhances GABPα binding to several SNPs in the ETS⬄CRE motif (30ng concentration). Same as in Figures 3 and S5, but using intensities obtained from ETS-CRE PBM experiments using 30ng concentrations of GABPα and CREB1.
