# Supplementary material for: GABPα Binding to Overlapping ETS and CRE DNA Motifs Is Enhanced by CREB1: Custom DNA Microarrays
Source: G3 (Bethesda). 2015 Jul 16;5(9):1909–18. doi: 10.1534/g3.115.020248 (PMC4555227; doi:10.1534/g3.115.020248)
Supplement: Supporting Information [file supp_g3.115.020248_FigureS7.pdf]

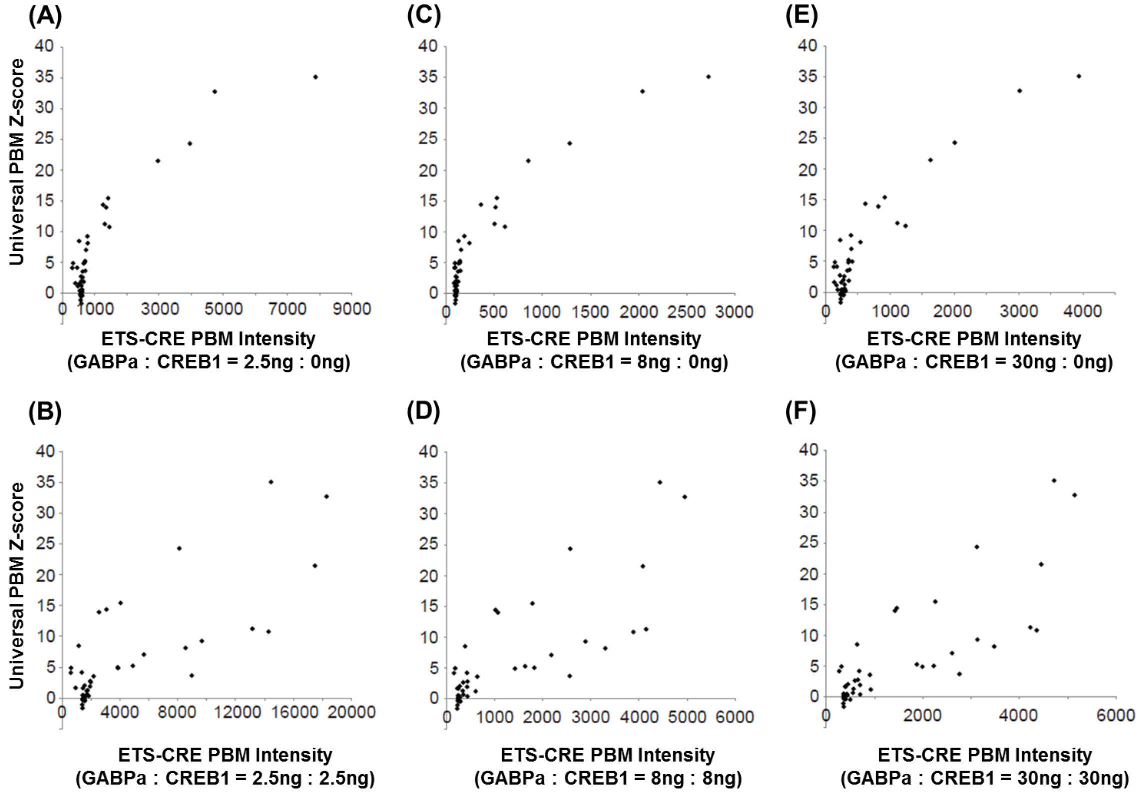

Figure S7 Comparison between universal and custom PBMs. Comparison of custom ETS-CRE array median signal intensity (X axis) and universal PBM Z-scores (Y axis) for each of 46 possible 8 base variants of the ETS motif. (A,C,E) Results using ETS-CRE arrays in the absence of CREB1. (A) 2.5ng GABPα, 0ng CREB1. (C) 8ng GABPα, 0ng CREB1. (E) 30ng GABPα, 0ng CREB1. (B,D,F) Results using ETS-CRE arrays in the presence of equal amounts of CREB1. (B) 2.5ng GABPα, 2.5ng CREB1. (D) 8ng GABPα, 8ng CREB1. (E) 30ng GABPα, 30ng CREB1.
