# Supplementary material for: GABPα Binding to Overlapping ETS and CRE DNA Motifs Is Enhanced by CREB1: Custom DNA Microarrays
Source: G3 (Bethesda). 2015 Jul 16;5(9):1909–18. doi: 10.1534/g3.115.020248 (PMC4555227; doi:10.1534/g3.115.020248)
Supplement: Supporting Information [file supp_g3.115.020248_FigureS8.pdf]

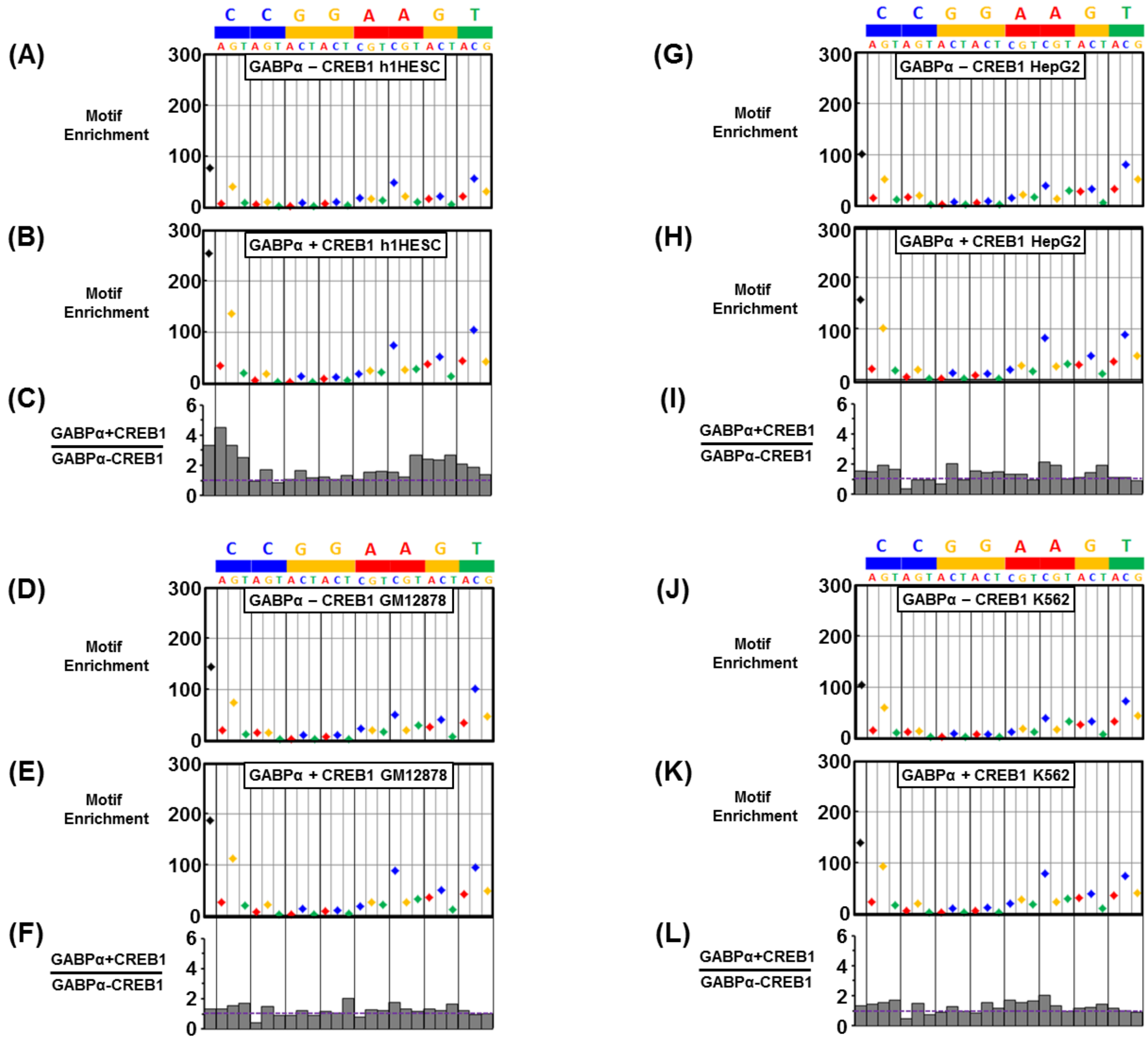

Figure S8 GABPα and CREB1 binding to SNPs in genomic regions co-bound by CREB1 and GABPα, in different cell lines. (A-B) Enrichment of consensus and each 1-bp variation of ETS motif (CCGGAAGT) in GABPα ChIP-seq peaks (A) that do not overlap with CREB1 ChIP-seq peaks (GABPα - CREB1), and (B) that do overlap with CREB1 ChIP-seq peaks (GABPα + CREB1), in h1HESC cells. (C) Histogram of the ratio of motif enrichment +/- CREB1 to the consensus and 1-bp variations of the ETS motif (CCGGAAGT) in h1HESC cells. (D-L) same as in A-C, but for: (D-F) GM12878 cells, (G-I) HepG2 cells, and (J-L) K562 cells.
