# Supplementary material for: GABPα Binding to Overlapping ETS and CRE DNA Motifs Is Enhanced by CREB1: Custom DNA Microarrays
Source: G3 (Bethesda). 2015 Jul 16;5(9):1909–18. doi: 10.1534/g3.115.020248 (PMC4555227; doi:10.1534/g3.115.020248)
Supplement: Supporting Information [file supp_g3.115.020248_TableS1.pdf]

Table S1 GABPα peaks with/without CREB1 peaks in 5 cell-lines

Cell-lines	All GABPα peaks			GAPBα + CREB1			GABPα – CREB1		
	#	Length	%Genome	#	Length	%Genome	#	Length	%Genome
A549	10,940	5,687,585	0.20%	5,776	1,443,503	0.05%	5,164	4,244,082	0.15%
GM12878	6,381	2,914,811	0.10%	5,298	2,001,359	0.07%	1,083	913,452	0.03%
H1hESC	5,596	2,454,341	0.09%	4,324	1,210,375	0.04%	1,272	1,243,966	0.04%
HepG2	9,776	4,137,716	0.14%	8,873	2,677,258	0.09%	903	1,460,458	0.05%
K562	14,112	5,299,216	0.19%	7,598	1,993,592	0.07%	6,514	3,305,624	0.12%
