# Supplementary material for: GABPα Binding to Overlapping ETS and CRE DNA Motifs Is Enhanced by CREB1: Custom DNA Microarrays
Source: G3 (Bethesda). 2015 Jul 16;5(9):1909–18. doi: 10.1534/g3.115.020248 (PMC4555227; doi:10.1534/g3.115.020248)
Supplement: Supporting Information [file supp_g3.115.020248_TableS2.pdf]

Table S2A GABPα binding to the consensus and 1bp SNPs of ETS motif in A549 (All Peaks)

ID	Base	Mer	#Genome	GABPα-CREB			GABPα+CREB			Fold increase	p-Value
				#	%	Enrich.	#	%	Enrich.		
0	N	CCGGAAGT	8,608	596	6.9%	46.68	1049	12.2%	241.56	5.17	2.41E-280
1	A	ACGGAAGT	9,033	133	1.5%	9.93	129	1.4%	28.31	2.85	7.10E-19
2	G	GCGGAAGT	6,579	241	3.7%	24.70	468	7.1%	141.01	5.71	2.07E-136
3	T	TCGGAAGT	6,562	64	1.0%	6.58	76	1.2%	22.96	3.49	3.87E-15
4	A	CAGGAAGT	88,465	1,106	1.3%	8.43	300	0.3%	6.72	0.80	4.95E-04
5	G	CGGGAAGT	9,785	153	1.6%	10.54	105	1.1%	21.27	2.02	1.57E-08
6	T	CTGGAAGT	73,134	253	0.3%	2.33	71	0.1%	1.92	0.83	1.52E-01
7	A	CCAGAAGT	67,739	228	0.3%	2.27	58	0.1%	1.70	0.75	4.75E-02
8	C	CCCGAAGT	7,646	73	1.0%	6.44	34	0.4%	8.81	1.37	1.28E-01
9	T	CCTGAAGT	64,674	179	0.3%	1.87	49	0.1%	1.50	0.80	1.77E-01
10	A	CCGAAAGT	6,499	45	0.7%	4.67	23	0.4%	7.02	1.50	1.10E-01
11	C	CCGCAAGT	5,106	41	0.8%	5.41	25	0.5%	9.71	1.79	1.96E-02
12	T	CCGTAAGT	7,499	32	0.4%	2.88	13	0.2%	3.44	1.19	5.89E-01
13	C	CCGGCAGT	7,894	104	1.3%	8.88	74	0.9%	18.58	2.09	6.89E-07
14	G	CCGGGAGT	9,408	166	1.8%	11.90	127	1.3%	26.76	2.25	1.59E-12
15	T	CCGGTAGT	2,777	40	1.4%	9.71	25	0.9%	17.84	1.84	1.54E-02
16	C	CCGGACGT	1,756	49	2.8%	18.81	81	4.6%	91.44	4.86	3.85E-22
17	G	CCGGAGGT	8,121	149	1.8%	12.37	92	1.1%	22.46	1.82	5.02E-06
18	T	CCGGATGT	6,008	132	2.2%	14.81	112	1.9%	36.95	2.49	1.76E-13
19	A	CCGGAAAT	7,627	174	2.3%	15.38	175	2.3%	45.48	2.96	2.15E-26
20	C	CCGGAACT	6,036	151	2.5%	16.87	168	2.8%	55.17	3.27	4.17E-29
21	T	CCGGAATT	5,550	53	1.0%	6.44	28	0.5%	10.00	1.55	5.74E-02
22	A	CCGGAAGA	10,630	249	2.3%	15.79	235	2.2%	43.82	2.77	1.07E-31
23	C	CCGGAAGC	11,970	509	4.3%	28.67	738	6.2%	122.21	4.26	1.25E-165
24	G	CCGGAAGG	12,065	361	3.0%	20.17	312	2.6%	51.26	2.54	6.88E-36

"Enrich." is the enrichment score for each motif (see also Method). P-Value is calculated based on Fisher's exact test.

Table S2B GABPα binding to the consensus and 1bp SNPs of ETS motif in K562 (All Peaks)

ID	Base	Mer	#Genome	GABPα-CREB			GABPα+CREB			Fold increase	p-Value
				#	%	Enrich.	#	%	Enrich.		
0	N	CCGGAAGT	8,608	1,043	12.1%	104.88	835	9.7%	139.23	1.33	9.34E-10
1	A	ACGGAAGT	9,033	161	1.8%	15.43	139	1.5%	22.09	1.43	1.84E-03
2	G	GCGGAAGT	6,579	451	6.9%	59.34	424	6.4%	92.50	1.56	3.66E-11
3	T	TCGGAAGT	6,562	73	1.1%	9.63	75	1.1%	16.40	1.70	1.04E-03
4	A	CAGGAAGT	88,465	1,211	1.4%	11.85	335	0.4%	5.44	0.46	2.43E-38
5	G	CGGGAAGT	9,785	146	1.5%	12.92	132	1.3%	19.36	1.50	6.88E-04
6	T	CTGGAAGT	73,134	191	0.3%	2.26	84	0.1%	1.65	0.73	1.54E-02
7	A	CCAGAAGT	67,739	158	0.2%	2.02	84	0.1%	1.78	0.88	3.50E-01
8	C	CCCGAAGT	7,646	70	0.9%	7.92	53	0.7%	9.95	1.26	2.11E-01
9	T	CCTGAAGT	64,674	132	0.2%	1.77	74	0.1%	1.64	0.93	6.15E-01
10	A	CCGAAAGT	6,499	46	0.7%	6.13	24	0.4%	5.30	0.87	5.65E-01
11	C	CCGCAAGT	5,106	44	0.9%	7.46	41	0.8%	11.52	1.55	4.34E-02
12	T	CCGTAAGT	7,499	20	0.3%	2.31	14	0.2%	2.68	1.16	6.69E-01
13	C	CCGGCAGT	7,894	103	1.3%	11.29	106	1.3%	19.27	1.71	9.28E-05
14	G	CCGGGAGT	9,408	190	2.0%	17.48	180	1.9%	27.46	1.57	1.19E-05
15	T	CCGGTAGT	2,777	36	1.3%	11.22	36	1.3%	18.61	1.66	3.01E-02
16	C	CCGGACGT	1,756	79	4.5%	38.94	96	5.5%	78.47	2.01	2.51E-06
17	G	CCGGAGGT	8,121	159	2.0%	16.95	128	1.6%	22.62	1.33	1.47E-02
18	T	CCGGATGT	6,008	220	3.7%	31.70	123	2.0%	29.38	0.93	5.01E-01
19	A	CCGGAAAT	7,627	233	3.1%	26.44	161	2.1%	30.30	1.15	1.84E-01
20	C	CCGGAACT	6,036	221	3.7%	31.69	163	2.7%	38.76	1.22	5.08E-02
21	T	CCGGAATT	5,550	43	0.8%	6.71	37	0.7%	9.57	1.43	1.11E-01
22	A	CCGGAAGA	10,630	387	3.6%	31.51	268	2.5%	36.19	1.15	8.17E-02
23	C	CCGGAAGC	11,970	1,000	8.4%	72.31	611	5.1%	73.26	1.01	8.00E-01
24	G	CCGGAAGG	12,065	607	5.0%	43.55	337	2.8%	40.09	0.92	2.23E-01

"Enrich." is the enrichment score for each motif (see also Method). P-Value is calculated based on Fisher's exact test.

Table S2C GABPα binding to the consensus and 1bp SNPs of ETS motif in HepG2

ID	Base	Mer	#Genome	GABPα-CREB			GABPα+CREB			Fold increase	p-Value
				#	%	Enrich.	#	%	Enrich.		
0	N	CCGGAAGT	8,608	445	5.2%	101.28	1262	14.7%	156.69	1.55	1.50E-15
1	A	ACGGAAGT	9,033	66	0.7%	14.32	182	2.0%	21.53	1.50	4.22E-03
2	G	GCGGAAGT	6,579	174	2.6%	51.82	618	9.4%	100.39	1.94	4.23E-15
3	T	TCGGAAGT	6,562	37	0.6%	11.05	111	1.7%	18.08	1.64	8.77E-03
4	A	CAGGAAGT	88,465	716	0.8%	15.86	452	0.5%	5.46	0.34	3.37E-77
5	G	CGGGAAGT	9,785	99	1.0%	19.82	174	1.8%	19.00	0.96	7.38E-01
6	T	CTGGAAGT	73,134	72	0.1%	1.93	125	0.2%	1.83	0.95	7.13E-01
7	A	CCAGAAGT	67,739	78	0.1%	2.26	97	0.1%	1.53	0.68	1.02E-02
8	C	CCCGAAGT	7,646	24	0.3%	6.15	89	1.2%	12.44	2.02	1.77E-03
9	T	CCTGAAGT	64,674	49	0.1%	1.48	87	0.1%	1.44	0.97	8.58E-01
10	A	CCGAAAGT	6,499	16	0.2%	4.82	46	0.7%	7.56	1.57	1.18E-01
11	C	CCGCAAGT	5,106	22	0.4%	8.44	57	1.1%	11.93	1.41	1.66E-01
12	T	CCGTAAGT	7,499	7	0.1%	1.83	19	0.3%	2.71	1.48	3.72E-01
13	C	CCGGCAGT	7,894	58	0.7%	14.39	140	1.8%	18.95	1.32	7.71E-02
14	G	CCGGGAGT	9,408	100	1.1%	20.82	247	2.6%	28.06	1.35	1.16E-02
15	T	CCGGTAGT	2,777	24	0.9%	16.93	41	1.5%	15.78	0.93	7.84E-01
16	C	CCGGACGT	1,756	34	1.9%	37.93	133	7.6%	80.95	2.13	5.36E-05
17	G	CCGGAGGT	8,121	56	0.7%	13.51	196	2.4%	25.79	1.91	1.41E-05
18	T	CCGGATGT	6,008	91	1.5%	29.68	171	2.8%	30.42	1.03	8.49E-01
19	A	CCGGAAAT	7,627	104	1.4%	26.72	213	2.8%	29.85	1.12	3.54E-01
20	C	CCGGAACT	6,036	101	1.7%	32.78	263	4.4%	46.57	1.42	2.58E-03
21	T	CCGGAATT	5,550	16	0.3%	5.65	57	1.0%	10.98	1.94	1.68E-02
22	A	CCGGAAGA	10,630	173	1.6%	31.89	360	3.4%	36.20	1.14	1.70E-01
23	C	CCGGAAGC	11,970	485	4.1%	79.38	987	8.2%	88.13	1.11	5.94E-02
24	G	CCGGAAGG	12,065	319	2.6%	51.80	520	4.3%	46.06	0.89	9.86E-02

"Enrich." is the enrichment score for each motif (see also Method). P-Value is calculated based on Fisher's exact test.

Table S2D GABPα binding to the consensus and 1bp SNPs of ETS motif in H1hESC

ID	Base	Mer	#Genome	GABPα-CREB			GABPα+CREB			Fold increase	p-Value
				#	%	Enrich.	#	%	Enrich.		
0	N	CCGGAAGT	8,608	285	3.3%	76.16	926	10.8%	254.31	3.34	1.19E-79
1	A	ACGGAAGT	9,033	29	0.3%	7.38	127	1.4%	33.24	4.50	1.08E-15
2	G	GCGGAAGT	6,579	117	1.8%	40.91	378	5.7%	135.83	3.32	2.28E-33
3	T	TCGGAAGT	6,562	23	0.4%	8.06	56	0.9%	20.17	2.50	1.26E-04
4	A	CAGGAAGT	88,465	197	0.2%	5.12	181	0.2%	4.84	0.94	5.78E-01
5	G	CGGGAAGT	9,785	44	0.4%	10.34	72	0.7%	17.39	1.68	6.01E-03
6	T	CTGGAAGT	73,134	70	0.1%	2.20	58	0.1%	1.87	0.85	3.65E-01
7	A	CCAGAAGT	67,739	53	0.1%	1.80	55	0.1%	1.92	1.07	7.38E-01
8	C	CCCGAAGT	7,646	25	0.3%	7.52	40	0.5%	12.37	1.64	4.87E-02
9	T	CCTGAAGT	64,674	44	0.1%	1.56	49	0.1%	1.79	1.14	5.15E-01
10	A	CCGAAAGT	6,499	18	0.3%	6.37	21	0.3%	7.64	1.20	5.71E-01
11	C	CCGCAAGT	5,106	23	0.5%	10.36	24	0.5%	11.11	1.07	8.11E-01
12	T	CCGTAAGT	7,499	11	0.1%	3.37	14	0.2%	4.41	1.31	5.04E-01
13	C	CCGGCAGT	7,894	59	0.7%	17.19	60	0.8%	17.97	1.05	8.10E-01
14	G	CCGGGAGT	9,408	65	0.7%	15.89	98	1.0%	24.63	1.55	5.78E-03
15	T	CCGGTAGT	2,777	16	0.6%	13.25	25	0.9%	21.28	1.61	1.35E-01
16	C	CCGGACGT	1,756	37	2.1%	48.47	55	3.1%	74.04	1.53	4.46E-02
17	G	CCGGAGGT	8,121	73	0.9%	20.68	88	1.1%	25.62	1.24	1.75E-01
18	T	CCGGATGT	6,008	27	0.4%	10.34	71	1.2%	27.94	2.70	4.64E-06
19	A	CCGGAAAT	7,627	52	0.7%	15.68	121	1.6%	37.50	2.39	5.75E-08
20	C	CCGGAACT	6,036	57	0.9%	21.72	132	2.2%	51.70	2.38	1.66E-08
21	T	CCGGAATT	5,550	12	0.2%	4.97	31	0.6%	13.20	2.66	2.81E-03
22	A	CCGGAAGA	10,630	96	0.9%	20.77	195	1.8%	43.37	2.09	1.57E-09
23	C	CCGGAAGC	11,970	290	2.4%	55.73	531	4.4%	104.87	1.88	1.32E-18
24	G	CCGGAAGG	12,065	158	1.3%	30.12	211	1.7%	41.34	1.37	2.51E-03

"Enrich." is the enrichment score for each motif (see also Method). P-Value is calculated based on Fisher's exact test.

Table S2E GABPα binding to the consensus and 1bp SNPs of ETS motif in GM12878

ID	Base	Mer	#Genome	GABPα-CREB			GABPα+CREB			Fold increase	p-Value
				#	%	Enrich.	#	%	Enrich.		
0	N	CCGGAAGT	8,608	394	4.6%	143.38	1,130	13.1%	187.68	1.31	3.91E-06
1	A	ACGGAAGT	9,033	57	0.6%	19.77	167	1.8%	26.43	1.34	5.73E-02
2	G	GCGGAAGT	6,579	155	2.4%	73.80	519	7.9%	112.79	1.53	3.04E-06
3	T	TCGGAAGT	6,562	24	0.4%	11.46	89	1.4%	19.39	1.69	2.06E-02
4	A	CAGGAAGT	88,465	409	0.5%	14.48	383	0.4%	6.19	0.43	7.27E-35
5	G	CGGGAAGT	9,785	45	0.5%	14.41	146	1.5%	21.33	1.48	2.05E-02
6	T	CTGGAAGT	73,134	43	0.1%	1.84	86	0.1%	1.68	0.91	6.25E-01
7	A	CCAGAAGT	67,739	44	0.1%	2.03	89	0.1%	1.88	0.92	6.65E-01
8	C	CCCGAAGT	7,646	25	0.3%	10.24	68	0.9%	12.72	1.24	3.54E-01
9	T	CCTGAAGT	64,674	37	0.1%	1.79	73	0.1%	1.61	0.90	6.03E-01
10	A	CCGAAAGT	6,499	14	0.2%	6.75	36	0.6%	7.92	1.17	6.11E-01
11	C	CCGCAAGT	5,106	16	0.3%	9.82	37	0.7%	10.36	1.06	8.57E-01
12	T	CCGTAAGT	7,499	4	0.1%	1.67	18	0.2%	3.43	2.05	1.83E-01
13	C	CCGGCAGT	7,894	59	0.7%	23.41	102	1.3%	18.47	0.79	1.47E-01
14	G	CCGGGAGT	9,408	60	0.6%	19.98	171	1.8%	25.99	1.30	7.88E-02
15	T	CCGGTAGT	2,777	15	0.5%	16.92	40	1.4%	20.59	1.22	5.16E-01
16	C	CCGGACGT	1,756	28	1.6%	49.95	109	6.2%	88.75	1.78	5.95E-03
17	G	CCGGAGGT	8,121	51	0.6%	19.67	148	1.8%	26.06	1.32	8.25E-02
18	T	CCGGATGT	6,008	55	0.9%	28.68	138	2.3%	32.84	1.15	3.95E-01
19	A	CCGGAAAT	7,627	64	0.8%	26.29	187	2.5%	35.05	1.33	4.61E-02
20	C	CCGGAACT	6,036	79	1.3%	41.00	207	3.4%	49.03	1.20	1.76E-01
21	T	CCGGAATT	5,550	13	0.2%	7.34	47	0.8%	12.11	1.65	1.06E-01
22	A	CCGGAAGA	10,630	115	1.1%	33.89	307	2.9%	41.29	1.22	7.03E-02
23	C	CCGGAAGC	11,970	387	3.2%	101.27	787	6.6%	94.00	0.93	2.30E-01
24	G	CCGGAAGG	12,065	182	1.5%	47.25	412	3.4%	48.82	1.03	7.14E-01

"Enrich." is the enrichment score for each motif (see also Method). P-Value is calculated based on Fisher's exact test.
